# Identification and prioritisation of potential vaccine candidates using subtractive proteomics and designing of a multi-epitope vaccine against *Wuchereria bancrofti*

**DOI:** 10.1038/s41598-024-52457-x

**Published:** 2024-01-23

**Authors:** Murali Aarthy, G. Navaneetha Pandiyan, R. Paramasivan, Ashwani Kumar, Bhavna Gupta

**Affiliations:** 1 ICMR-Vector Control Research Centre (VCRC), Field Station, Madurai, Tamil Nadu 625002 India; 2 ICMR-Vector Control Research Centre (VCRC), Puducherry, India; 3 Present Address: Saveetha Institute of Medical and Technical Sciences (SIMATS), Saveetha University, Tandhalam, Chennai, Tamil Nadu 602105 India

**Keywords:** Bioinformatics, Data acquisition, Data processing, Protein structure predictions, Proteome informatics

## Abstract

This study employed subtractive proteomics and immunoinformatics to analyze the *Wuchereria bancrofti* proteome and identify potential therapeutic targets, with a focus on designing a vaccine against the parasite species. A comprehensive bioinformatics analysis of the parasite's proteome identified 51 probable therapeutic targets, among which "Kunitz/bovine pancreatic trypsin inhibitor domain-containing protein" was identified as the most promising vaccine candidate. The candidate protein was used to design a multi-epitope vaccine, incorporating B-cell and T-cell epitopes identified through various tools. The vaccine construct underwent extensive analysis of its antigenic, physical, and chemical features, including the determination of secondary and tertiary structures. Docking and molecular dynamics simulations were performed with HLA alleles, Toll-like receptor 4 (TLR4), and TLR3 to assess its potential to elicit the human immune response. Immune simulation analysis confirmed the predicted vaccine’s strong binding affinity with immunoglobulins, indicating its potential efficacy in generating an immune response. However, experimental validation and testing of this multi-epitope vaccine construct would be needed to assess its potential against *W. bancrofti* and even for a broader range of lymphatic filarial infections given the similarities between *W. bancrofti* and *Brugia*.

## Introduction

Lymphatic filariasis is a neglected tropical disease that poses a significant public health burden worldwide. It is primarily transmitted through the bites of infected mosquitoes carrying parasites of the *Wuchereria bancrofti*, *Brugia malayi*, and *B. timori* species. Among these, *W. bancrofti* accounts for more than 90% of lymphatic filariasis cases globally^1,2^. The impact of lymphatic filariasis on affected individuals and communities is severe. The disease causes chronic and debilitating conditions such as lymphedema, elephantiasis, and hydrocele^3^. These conditions not only lead to physical disability but also result in social stigma, economic hardship, and reduced productivity. The burden of the disease falls disproportionately on marginalized populations in low-resource settings, further exacerbating health inequities.

To address this global health challenge, the World Health Organization (WHO) launched the Global Program to Eliminate Lymphatic Filariasis (GPELF) in 2000. The program aimed to eliminate lymphatic filariasis as a public health problem by 2020^4^ and this timeline for elimination has been extended to 2030 now^5^. GPELF adopted a comprehensive approach that combined mass drug administration (MDA) with other preventive measures such as vector control, morbidity management, and disability prevention. MDA involves the administration of anti-filarial drugs, including Albendazole, Ivermectin, and Diethylcarbamazine (DEC)^6^, to entire at-risk populations in endemic areas. These drugs target the microfilariae, the larval stage of the parasites circulating in the bloodstream, and aim to reduce their numbers, prevent transmission, and alleviate symptoms. The success of MDA campaigns in reducing disease burden has been significant, and several countries have made remarkable progress toward elimination goals^7,8^. However, challenges remain on the path toward complete elimination of lymphatic filariasis. One of the primary challenges is the persistence of adult worms in infected individuals, as the currently available drugs primarily target the microfilariae^9,10^. These adult worms can continue to produce microfilariae, perpetuating the transmission cycle of the disease. Additionally, there are concerns about the emergence of drug resistance in the parasite populations^11–16^. There have been some reports of poor responses to DEC^12,13,16^ treatment as well as to Ivermectin^11,17^. Moreover, a mutation associated with resistance to Albendazole has been detected in *W. bancroft*i populations^15^. Such challenges could impact the effectiveness of MDA interventions. Innovative strategies are required to overcome these challenges and achieve sustained elimination. One promising approach is the development of vaccines against lymphatic filariasis. Vaccines have the potential to provide long-lasting protection, target multiple stages of the parasite's life cycle, and complement existing control measures^18–20^. Extensive research efforts have been dedicated to identifying suitable vaccine candidates using various approaches, including subtractive genomics and reverse vaccinology^21–24^.

Subtractive genomics involves comparing the genomes of the parasite with the host organisms to identify unique proteins or metabolic pathways that could be potential targets for interventions. This approach helps in prioritizing proteins essential for the survival or reproduction of the parasite^25^, that could be explored to develop potential vaccines or new drug therapies. Reverse vaccinology complements subtractive genomics to predict immunogenic epitopes, design and evaluate multi-epitope vaccines^26,27^ using various immune-informatics tools. This computational approach aids in the selection of vaccine candidates with the highest potential for success, minimizing the need for extensive experimental testing^28^.

Furthermore, significant advancements in high-throughput sequencing technologies and proteomics have facilitated the identification and characterization of potential vaccine targets. By analyzing the proteome of the parasite, the proteins involved in host-parasite interactions, immune evasion mechanisms, and pathogenesis can be identified^29^. This knowledge guides the development of effective vaccines that can stimulate robust and specific immune responses against parasites^30,31^. Several potential vaccine targets have been identified and characterized in *B. malayi* using these innovative approaches. Some of the promising vaccine candidates for combating lymphatic filariasis include abundant larval transcript 2 (ALT-2)^32^, thioredoxin peroxidase (TPX)^32^, vespid venom allergen (VAH)^33^, heat shock protein (HSP)^34^, glutathione S-transferases (GST)^35^. However, these antigens have not yielded satisfactory results individually. Combining these candidates into multivalent vaccines has shown potential in addressing lymphatic filariasis. For instance, recombinant multivalent fusion protein vaccine (rBmHAXT) the combination of ALT-2, HSP, thioredoxin peroxidase 2, and tetraspanin large extracellular loop is among the most promising candidates. It has demonstrated 88–94% protection against challenge infections in rodents^36,37^ and approximately 57% protection in rhesus macaques^38^. Another multivalent vaccine candidate is the combination of *B. malayi* VAH and ALT-2^39^. Further expanding and diversifying the database of potential vaccine targets is essential for advancing vaccine development efforts in the fight against lymphatic filariasis.

In this study, subtractive proteomics and reverse vaccinology techniques were employed to identify potential vaccine candidates for lymphatic filariasis by screening the putative proteome of *W. bancrofti.* Through prioritization of the proteins with anti-parasitic properties, the study provides valuable insights into promising targets that could be explored further for their vaccine development potential. The protein named “Kunitz type inhibitor domain-containing protein” (VDM15541), emerged as the most promising vaccine candidate among the 51 therapeutic targets identified. This protein was further analyzed to design a multi-epitope vaccine construct. Extensive analysis of the antigenic, physical, and chemical features of the vaccine construct, including its secondary and tertiary structure, confirmed its potential immunogenicity. Multi-epitope vaccines contain multiple epitopes or antigenic regions from a pathogen's proteins. Unlike traditional single-antigen vaccines, multi-epitope vaccines provide several advantages. Multiple epitopes enhance the immune response and reduces the chances of immune escape as well as development of resistance in the pathogens^40,41^. Multi-epitope vaccines hold great promise for the development of effective and versatile vaccine solutions.

## Materials and methods

### Data retrieval

The entire set of 13,058 proteins linked to *W. bancrofti* Genome Project PRJEB536 with the Taxonomy ID: 6293 (BioSample: SAMEA1686771) was obtained from the NCBI database in January 2022. The human proteome dataset with the Genome Assembly ID: GrCh38.p14 was retrieved from NCBI.

### Subtractive proteomic analysis to identify and prioritize the potential vaccine candidates

**Figure 1:**
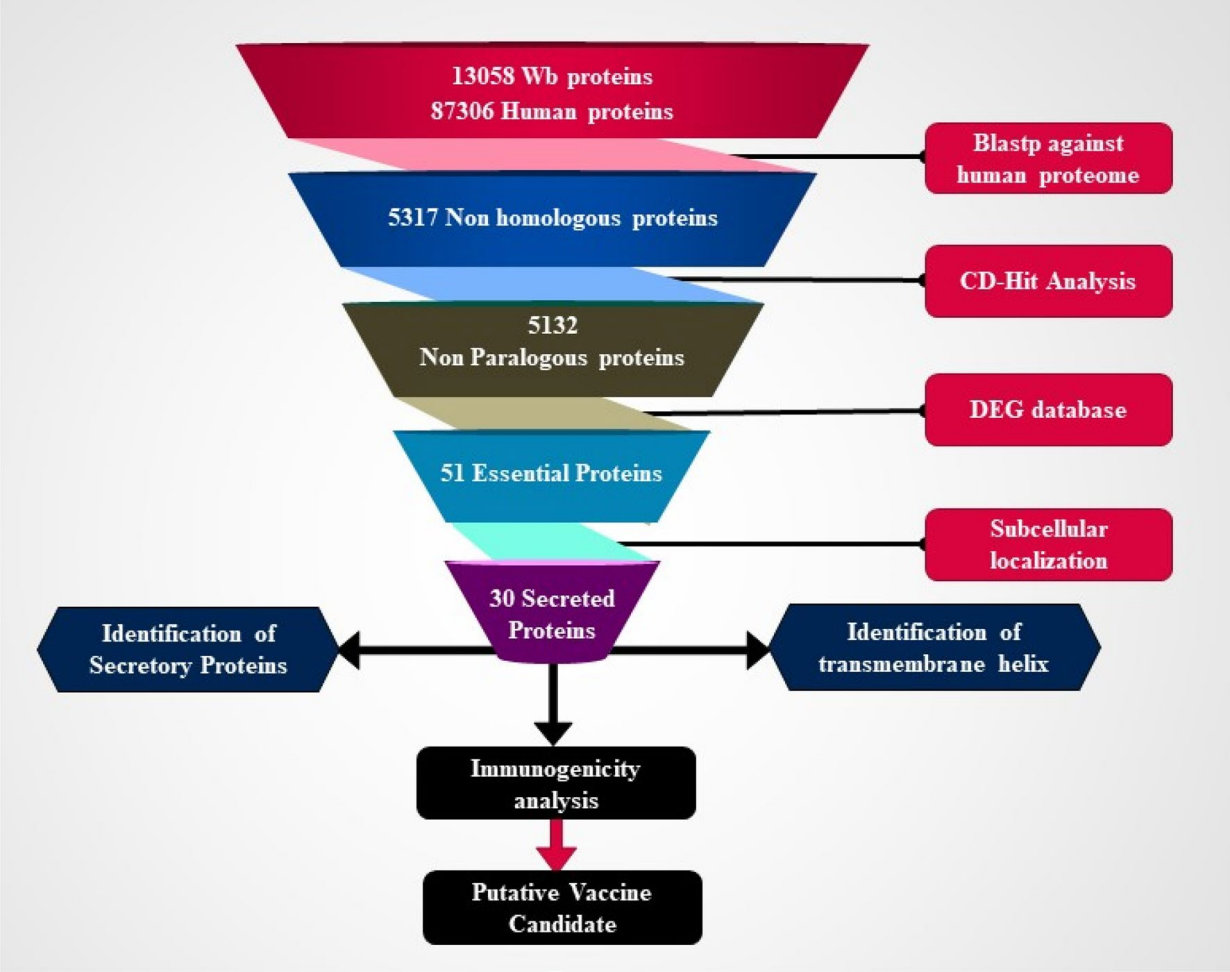
Workflow of subtractive proteomics to identify and potential vaccine candidates against *Wuchereria bancrofti.*

Subtractive proteomics helps to identify potential proteins with specific features from a large dataset using several subtractive steps as shown in Fig. 1. *W. bancrofti* proteome was BLAST analyzed against the human proteome to identify proteins with similarity. Proteins showing any similarity to human proteins were excluded from further analysis. CD-HIT analysis (https://www.bioinformatics.org/cd-hit/) was performed using a 75% similarity threshold to eliminate paralogous proteins and reduce redundancy. The remaining non-homologous and non-paralogous proteins were selected for further analysis. Using BLAST analysis, the selected proteins were compared to the Database of Essential Genes (DEG)^42^. Essential proteins were identified based on stringent criteria such as an e-value of 10^–10^, bit score 100, and percent identity of > 30. Although this strict approach may have excluded some potentially valuable information, the selected genes ensure high-quality candidates for further analysis. The subcellular localization of the identified proteins was predicted using the WoLF PSORT^43^ bioinformatics tool. Proteins predicted to be membrane bound or extracellular in nature were considered potential vaccine targets. The presence of a signal peptide, indicative of potential protein secretion, was analyzed using the SignalP 5.0 tool^44^. The SecretomeP tool^45^ was used to predict proteins potentially exported through non-classical pathways. Proteins predicted to be secretory through both classical and non-classical pathways were considered secreted proteins. Furthermore, the transmembrane domains in each protein were identified using the TMHMM server (https://services.healthtech.dtu.dk/services/TMHMM-2.0/)^46^. Proteins with more than one transmembrane helix were considered membrane-bound and excluded from further analysis. The VaxiJen v2.0^47^ and AntigenPro^48^ tools were utilized to assess the antigenicity and immunogenicity of the selected proteins, respectively. Proteins with high antigenicity and immunogenicity scores were prioritized as potential vaccine targets. Various bioinformatics analyses, such as sequence alignment, and domain analysis, were performed to further characterize the selected vaccine target.

### Reverse vaccinology to develop a putative vaccine against *W. bancrofti*

**Figure 2:**
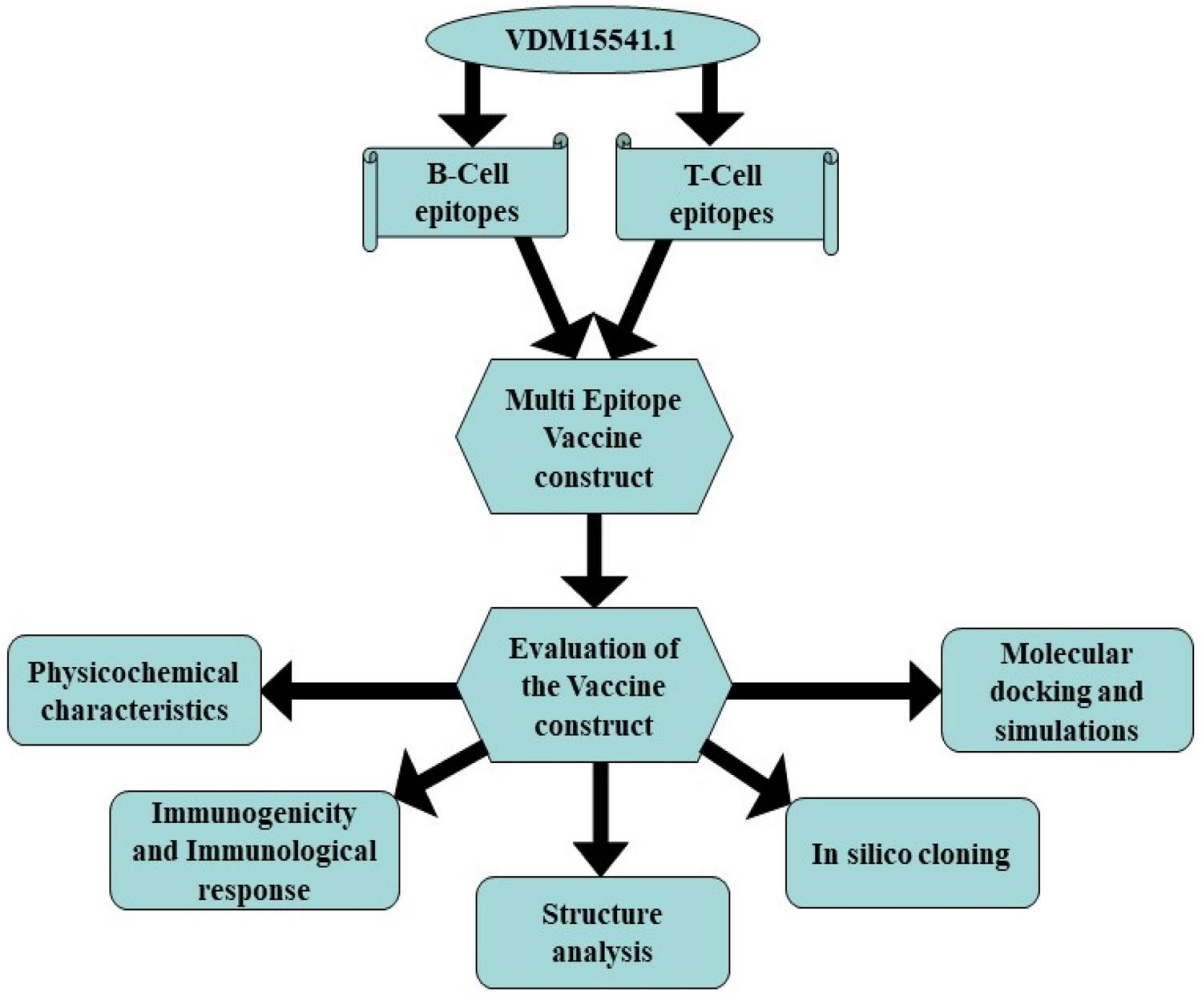
Workflow of reverse vaccinology analysis to design and evaluate multi-epitope vaccine against *Wuchereria bancrofti.*

Further analysis of the potential vaccine candidate was performed using immunoinformatics analysis (Fig. 2). MHC-I binding epitopes were predicted using the NetCTL server^49^ with a threshold for epitope identification of 0.75, weight on TAP transport efficiency 0.05 and weight on C terminal cleavage of 0.15. Immune Epitope Database Analysis Resource (IEDB AR)^50^ was used to predict the binding of shortlisted epitopes with MHC-1. MHC-II binding epitopes were predicted through IEDB 2023.05. B-cell epitopes were predicted using ABCPred^51^, BCePred prediction server^52^, and Bepipred 2.0^53^. Potent B-cell and MHC-I epitopes were combined to form a multi-epitope vaccine construct. Adjuvant β defensin was added at the N-terminal end and linked to MHC-I epitopes using the EAAAK linker. MHC-I epitopes were joined using the EAAAK linker, and B-cell epitopes were joined using the GPGPG linker.

The physical and chemical properties of the vaccine construct, including molecular weight, aliphatic index, instability index, GRAVY score, and solubility, were determined using ProtParam tool (https://web.expasy.org/protparam/protpar-ref.html).

### Evaluation of immune response of the vaccine construct

Antigenicity and immunogenicity of the vaccine construct were assessed using AntigenPro and VaxiJen v2.0, respectively. The allergic response of the vaccine construct was determined using AlgPred server^54^ and AllerCatPro server 2.0^55^.

### Prediction of post-translational modifications (PTM)s

The prediction of post-translational modifications including glycosylation, phosphorylation, and acetylation of the vaccine construct was carried out using NetNGlyc 1.0, NetPhos 3.0, and NetAct 1.0 servers, which are available at http://www.cbs.dtu.dk/services. Additionally, N-terminal glycines myristoyl and GPI-modifications were assessed using MyrPS/NMT (https://mendel.imp.ac.at/myristate/)^56^ and big-PI/GPI servers (https://mendel.imp.ac.at/gpi/gpi_server.html)^57^, respectively.

### Vaccine model construction

To design and construct the model of the vaccine, the sequence VDM15541 was retrieved from the UniProtKB database^58^. The sequence was checked for suitable template through BLAST using default parameters. The protein with the query coverage of 24% and percent identity of 95.56% was obtained. The length of the residues for template protein was only 45 when the length of query protein was 165 amino acids in length. Since, the query coverage was very low, we tried ab initio threading of the protein through i-TASSER-MTD^59^ and obtained five models. The five models were analyzed and validated through PDBSum generated for the Ramachandran plot analysis^60^. The best model was further used for the interaction studies with the human TLR3 and TLR4 protein^61,62^ and HLA alleles.

### Protein–protein docking

The best model of the vaccine construct was used for the docking with TLR3 encompassing the PDB ID: 1ZIW and TLR4 with the PDB ID: 3FXI in HDock (http://hdock.phys.hust.edu.cn/) along with the HLA alleles. The HDock server is a highly integrated suite of molecular modeling techniques. This includes homology search, template-based modeling, structure prediction, docking etc., This docking platform is different from other docking platforms which uses the amino acids as input and the strategy of docking in which the information about the binding site is retrieved from the experimental studies. Moreover, the small angle X-ray scattering is incorporated during the docking and post docking processes. HDock supports the docking function through the intrinsic scoring function^63,64^. Further, the binding affinity of the protein and alleles was calculated through the PPI binding tool (https://protdcal-suite.cbe.bci.tu-dortmund.de/PPIAffinity/). This tool uses binding affinity predictors from support vector machines to screen datasets of protein–protein and protein-peptide complexes ^65^.

### Molecular dynamics simulation

Molecular dynamics simulation was carried out with the help of MD Simulation tool incorporated in SiBIOLEAD which uses GROMACS simulation application (https://sibiolead.com/MDSIM). This web server uses five steps which includes preprocessing, energy minimization, equilibration, production dynamics and trajectory analysis. The protein complex of vaccine and TLR and HLA alleles were used for the simulation process. The protein complex was placed in a Cubic box filled with an SPC water model. Further, the protein complex was neutralized with the NaCl ions with the concentration of 0.15 molar. The steepest descent integrator with the 5000 steps was applied for the energy minimization parameters. The default equilibration type NVT/NPT with the temperature of 300 K, pressure of 1 bar with 100 ps was performed before the production dynamics in order to equilibrate the system. Finally, the Leapfrog integrator with the time period of 10 ns was carried out for the production dynamics with the 5000 frames^66^.

### In-silico cloning and codon optimisation

The JCat web server^67^ was used for back-translation and codon optimization of the vaccine construct. Codons were modified based on the codon usage preferences of the target host, *E. coli* K12, to enhance expression rates. The modified sequence was analyzed for GC content and codon adaptation index using the JCat webserver.

### Simulations for evaluating the immune response of the vaccine construct in host cells

The C-ImmSim server^68^ was used to simulate the immune response to the predicted vaccine construct. Three doses of the vaccine construct were administered at three-month intervals. The simulation tracked the levels of IgM, IgG1, and IgG2 antibodies over time to assess the immune response. The frequency and activation of CD8+ and CD4+ T-cells, as well as the formation of memory cells, were also monitored. Cytokine levels during the immune reaction were analyzed using the Simpson index D graph to evaluate potential risks and safety of the vaccine construct.

## Results and discussion

In this study, we aimed to identify potential vaccine targets against *W. bancrofti* by screening its entire proteome. Total 13,058 proteins were retrieved from the PRJEB536 genome sequence project. To ensure these targets would not induce adverse immunogenic effects by cross-reacting with human proteins, BLAST analysis was conducted against the human proteome, to exclude any similar proteins. Subsequently, the non-homologous proteins were analyzed using CD-HIT analysis (75% similarity threshold) to remove redundancy, resulting in 5132 proteins. We further subjected these proteins to BLAST analysis against the DEG database to identify essential proteins crucial for pathogen survival. A subset of 51 essential proteins (Table 1) was identified, which were selected based on stringent criteria. These 51 proteins represent promising candidates for further analysis in vaccine development against *W. bancrofti*, offering potential therapeutic targets. Further analysis of these 51 proteins was conducted to assess their immunogenicity, efficacy, and safety, providing valuable insights into their potential as vaccine candidates against *W. bancrofti*.

**Table 1:** List of 51 probably therapeutic targets identified from the analysis of *Wuchereria bancrofti* proteome.

Sl no.	Gene ID	Sub-cellular location	Signal IP	Secretome	TMHMM	VaxiJen Score
1	VDM06647.1	Plasma membrane			0	0.331
2	VDM06730.1	Plasma membrane			1	0.5839
3	VDM06817.1	Plasma membrane			6	0.5399
4	VDM06914.1	Plasma membrane			10	0.6464
5	VDM06935.1	Plasma membrane		Yes	12	0.4645
6	VDM07450.1	Plasma membrane			11	0.4436
7	VDM07534.1	Extracellular		Yes	3	0.6416
8	VDM09497.1	Plasma membrane			1	0.6711
9	VDM09927.1	Cytosol			0	0.5954
10	VDM09984.1	Plasma membrane			3	0.6092
11	VDM10251.1	Plasma membrane		Yes	1	0.3749
12	VDM10465.1	Cytosol			0	0.5102
13	VDM10950.1	Extracellular		Yes	4	0.4195
14	VDM10964.1	Cytosol			0	0.7157
15	VDM11313.1	Plasma membrane			7	0.574
16	VDM11729.1	Plasma membrane			4	0.6573
17	VDM12133.1	Plasma membrane			3	0.5382
18	VDM12167.1	Cytosol			0	0.2702
19	VDM12641.1	Cytosol			0	0.3612
20	VDM12677.1	Plasma membrane			2	0.5437
21	VDM12793.1	Plasma membrane			0	0.6694
22	VDM13458.1	Cytosol			0	0.7542
23	VDM13776.1	Plasma membrane			2	0.5851
24	VDM14680.1	Cytoskeleton			5	0.5977
25	VDM14801.1	Plasma membrane	Yes	Yes	3	0.6152
26	VDM15121.1	Extracellular			0	0.5909
27	VDM15129.1	Cytosol			0	0.4969
28	VDM15521.1	Nuclear			0	0.6647
29	VDM15541.1	Plasma membrane	Yes	Yes	1	0.8248
30	VDM15698.1	Cytosol			0	0.5138
31	VDM16641.1	Cytoskeleton			0	0.5628
32	VDM16965.1	Extracellular			0	0.1302
33	VDM17544.1	Extracellular			0	0.7654
34	VDM17973.1	Cytosol			1	0.6007
35	VDM19390.1	Cytosol			0	0.3326
36	VDM19792.1	Plasma membrane			2	0.6621
37	VDM20216.1	Cytoskeleton			3	0.5835
38	VDM20372.1	Plasma membrane			4	0.6906
39	VDM20417.1	Plasma membrane		Yes	4	0.4638
40	VDM20455.1	Mitochondria			0	0.401
41	VDM20976.1	Plasma membrane	Yes	Yes	12	0.6795
42	VDM21381.1	Cytosol			0	0.487
43	VDM21514.1	Extracellular			0	0.4478
44	VDM21712.1	Plasma membrane			10	0.6351
45	VDM21861.1	Cytosol			0	0.4379
46	VDM21901.1	Plasma membrane			3	0.557
47	VDM21936.1	Plasma membrane			9	0.5519
48	VDM22966.1	Endoplasmic reticulum			0	0.4424
49	VDM23249.1	Cytosol			0	0.515
50	VDM23299.1	Mitochondria			0	0.4192
51	VDM23483.1	Mitochondria			0	0.3879

### Prioritization of putative vaccine targets

To prioritize the putative vaccine targets several factors were taken into consideration. Firstly, the subcellular localization of the 51 identified proteins was determined using the WoLF PSORT bioinformatics tool. It was observed that 24 proteins were associated with the plasma membrane, six were extracellular, five were in the organelles, and 13 were in the cytosol (Table 1). Theoretically, the membrane-associated proteins are more exposed to the human immune system and are thus considered potential vaccine targets. Cytoplasmic membrane proteins are also important for the physiology of bacteria, as they are involved in many important metabolic functions and thus are potential drug targets. Out of the 51 proteins, 30 proteins (associated with plasma-membrane and extracellular) were found as the potential vaccine targets based on this analysis. However, as these predictions are based on bioinformatics algorithms, it is crucial to corroborate the findings with multiple analyses. For example, the secretory potential of the proteins was assessed using several methods. First, SignalP tool was used to identify the signal peptide, which facilitates transport to the extracellular region. Among the 51 proteins, only three were found to have a signal peptide (Table 1), suggesting their potential for secretion. Secondly, SecretomeP tool was employed using a mammalian secreted proteins database to identify the proteins which are likely to be exported through non-classical pathways that do not require a signal peptide^69^. This method detected four proteins (Table 1). Thirdly, the presence of transmembrane hydrophobic α-helices was determined using TMHMM server. The secretory proteins can have diverse locations, including the periplasm, outer membrane, or periplasmic side of the inner membrane. Membrane-bound proteins typically contain transmembrane hydrophobic α-helices which helps in their embedding in the membrane. However, membrane-bound proteins are difficult to purify^70,71^. The proteins with more than one transmembrane helix were considered membrane-bound and were excluded from further analysis. Among the analyzed 51 proteins, two proteins, namely VDM10251 and VDM15541, met the criteria of being secretory either having signal peptide or through the non-secretory pathway and having less than one transmembrane helix (Table 1). However, it is important to note that VDM10251 is a collagen protein typically found in the extracellular matrix, and it does not exhibit secretory properties. While protein VDM15541 is an orthologue of mlt-11 in *C. elegans* which is a secretory protein and is associated with molting^72^. VDM15541 also showed high antigenicity (0.825) and immunogenicity (0.943) as assessed by AntigenPro and VaxiJen tools, respectively.

Through this comprehensive analysis combining subcellular localization, secretion prediction, membrane localization, and antigenicity assessment, VDM15541, a 165 amino acid long protein was identified as the most promising vaccine candidate for further evaluation as a potential vaccine target against *W. bancrofti*.

### Importance of the selected vaccine target

Due to incomplete annotation of the *W. bancrofti*, further analysis of the identified protein (VDM15541) was conducted by comparing it to known proteins from *Brugia* and other nematodes. While there has not been an in-depth analysis of inter- and intra-species variations in the genome of lymphatic filariasis (LF) parasites, data comparing selected genes between *W. bancrofti* and *B. malayi* have demonstrated significant homology, with over 95% similarity^73^. The first 100 hits for VDM15541 from BLAST analysis are given in Table S1. The protein showed similarity with several nematodes including *Brugia, Caenorhabditis, Dirofilaria, Onchocerca, Loa loa* with percent identity ranging from 40 to 89% and query coverage from 26 to 95%. VDM15541 showed 80.13% identity with 92% query coverage with *B. malayi* Kunitz/Bovine pancreatic trypsin inhibitor domain-containing protein (BMA-MLT-11). BMA-MLT-11 is a secretory protein and has three known isoforms (Bm6109a, Bm6109b and Bm6109c). These proteins have not been characterized extensively. However, its orthologue mlt-11 in *C. elegans* is known to be associated with molting^72^. Knockdown of mlt-11 by RNAi induced Larval (L1) arrest and defects during larval molting^74–76^. *W. bancrofti* protein covered only a part of the BMA-MLT-11 (isoform Bm6109a) which is ~ 3000 amino-acid long. However, 100% similar protein was identified in all the four BioSamples sequenced under three different BioProjects (PRJNA526, PRJNA37759 and two samples under PRJNA275548) (Figure S1). The InterPro analysis of VDM15541 provided valuable insights into the protein's domain and other features (Figure S2). Notably, a pancreatic trypsin inhibitor Kunitz domain from 21 to 105 amino-acid was identified. Kunitz inhibitors have been recognized as promising vaccine targets, as they play a crucial role in modulating host immunity^77–79^. It is worth noting that Kunitz inhibitors have already shown promise as vaccine candidates in other helminths, such as *Fasciola hepatica* and *Schistosoma mansoni*^78,80–83^. Notably, Kunitz protease inhibitor gene Smp_147730 in *S. mansoni is* 146 amino-acid long and contains only one Kunitz/bovine protease inhibitor domain^81^ similar to VDM15541.

Kunitz-type inhibitors play a crucial role in the context of host blood feeding and pathogen transmission, particularly in vector-borne diseases^77^. For example, these inhibitors are often produced by blood-feeding arthropods, such as mosquitoes, ticks, and sandflies, as well as by the pathogens they transmit. When a blood-feeding arthropod bites a host, it injects saliva containing various bioactive molecules, including Kunitz-type inhibitors. These inhibitors are essential for the arthropod's successful blood meal acquisition by modulating host hemostasis and immune responses^84^. By inhibiting host proteases involved in blood clotting and inflammation, Kunitz-type inhibitors prevent the formation of blood clots and suppress host immune reactions at the bite site. This allows the arthropod to feed on blood more efficiently and remain undetected by the host's immune system.

In the context of pathogen transmission, Kunitz-type inhibitors have additional implications. They can interfere with the host's immune response to the pathogen, aiding in its establishment and survival within the host^82^. Interestingly, several helminths themselves produce Kunitz-type inhibitors as a means to manipulate the host's immune system. These pathogen-derived inhibitors can modulate host immune responses, hinder immune cell activation, and suppress immune defenses, ultimately promoting pathogen survival and dissemination within the host^82^.

Given the critical role of Kunitz-type inhibitors in host blood feeding and pathogen transmission, they have emerged as potential targets for intervention strategies. By targeting these inhibitors, it may be possible to disrupt the delicate balance between the vector, pathogen, and host interactions. However, it is important to note that further research is needed to fully understand the complexity of these interactions and the potential of targeting Kunitz-type inhibitors as a control strategy.

### Evaluation of selected protein as a vaccine target

#### Identification of T-cell and B-cell epitopes

The identification of T-cell and B-cell epitopes is crucial for designing an effective vaccine. T-cells are classified as CD4+ (HTLs) or CD8+ (CTLs) based on the receptors present on their membranes. These cells interact with epitopes presented by MHC-I and MHC-II molecules. CTLs bind to MHC-I binding epitopes, while HTLs interact with MHC-II molecules. Strong interactions between antigenic epitopes and MHC molecules lead to a robust immune response. Therefore, predicting high-affinity epitopes is important.

NetCTL identified 26 MHC ligands with a threshold score of 0.75 for epitope identification that gives 65% sensitivity and 97% specificity. Out of these 26 predicted epitopes, six showed binding to MHC_I alleles (Table S2). However, none of the MHC-II binding epitopes with a percentile rank below 0.2 were identified. Thus only MHC-I epitopes were further assessed for their antigenicity, conservancy, and toxicity. All the epitopes were found 100% conserved and non-toxic to the host cells. Out of six epitopes, four had antigenicity > 0.5 and thus those epitopes were selected for reverse vaccinology analysis (Table S2).

B-cell epitopes were predicted using servers ABCPred, BCePred, and BepiPred. ABCpred, employs an Artificial Neural Network (ANN) based method that considers amino acid sequences and their physicochemical properties to predict B cell epitopes. It calculates a propensity score for each amino acid in the sequence and subsequently assigns a binary classification (epitope or non-epitope). Threshold value of 0.5 and length of linear B-cell epitopes of 16 were used for epitope prediction on ABCpred server. Similarly, predicted residue score of 0.2 was used for BepiPred-2.0 which identifies the B cell epitopes based on a random forest algorithm trained on epitopes annotated from antibody-antigen protein structures. The ABCPred server predicted 12 B-cell epitopes, BepiPred identified seven epitopes and BCePred predicted six epitopes (Table S3), with the default threshold values for hydrophilicity, flexibility, accessibility, turns, exposed surface, polarity and antigenic propensity of polypeptides chains. On comparing all the epitopes six of them were identified as overlapping in two or more methods and were used for further analysis (Table 2).

**Table 2:** MHC-1 and B-cell epitopes selected for designing multi-epitope vaccine.

S. no.	MHC-I	B-cell epitopes
1	TTIVVAIEK	NDVNVCKRQPFRGRCPSVGGKGPA
2	ANDENEPVL	CVSY
3	RSQFVLRYY	FGHCANDENEPVLYR
4	ALLAQVAFV	LNKWDTNDTTIQSTTIQNDNEMEKSENSS
5		ITDNDNDSDSD
6		STTLSSSSFPIIDNNNDNNDT

#### Designing and characterization of a multi-epitope vaccine construct

The shortlisted potent B-cell and MHC-I epitopes were linked together to form a putative vaccine construct. An adjuvant, β-defensin, comprising 45 amino acids, was introduced at the N-terminal end of the vaccine construct. This adjuvant was connected to MHC-I alleles using the EAAAK linker. To enhance the overall effectiveness of the vaccine, MHC-I epitopes were linked together utilizing the EAAAK linker, while MHC-I alleles were connected to each other using the GPGPG linker. The final construct contained 182 amino acids with a molecular weight of 19 kDa.

**Figure 3:**
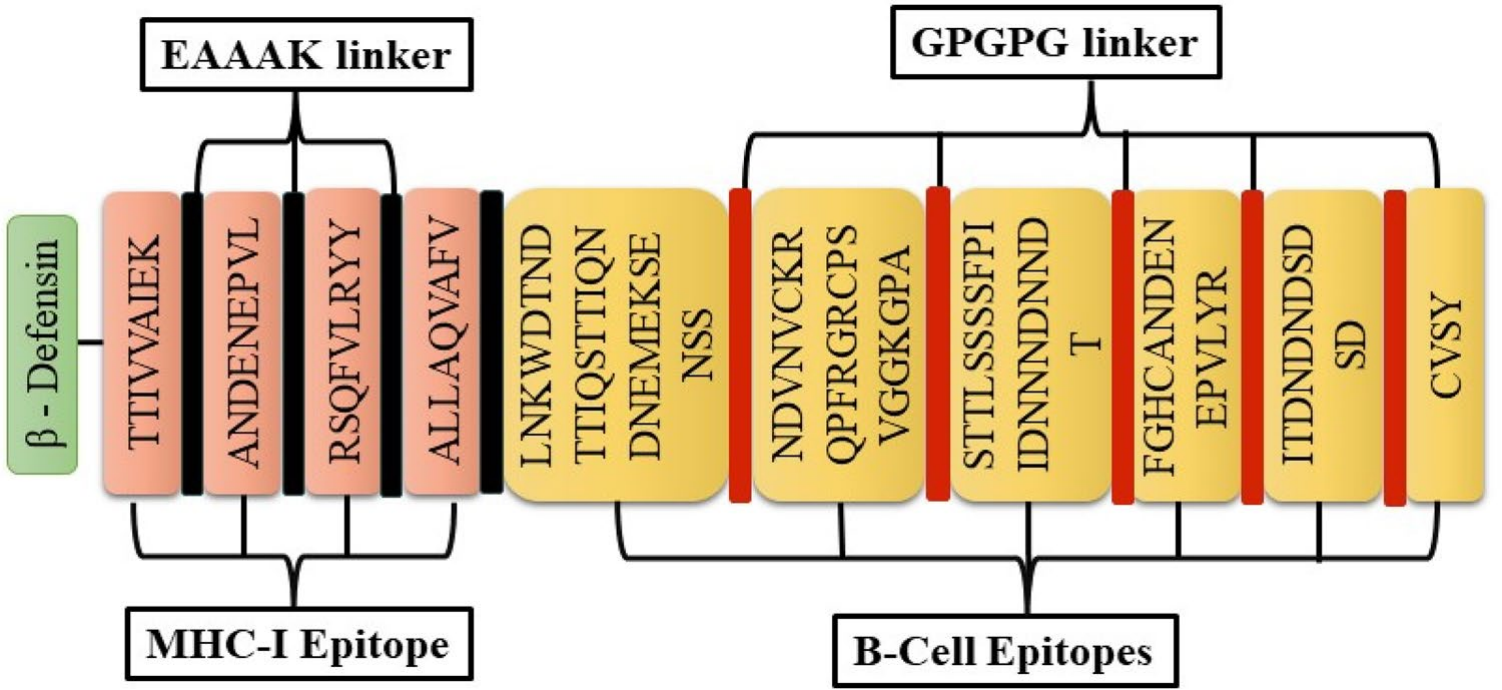
Schematic representation of the multi-epitope vaccine construct.

An important aspect to consider when evaluating a vaccine candidate is it’s immunogenicity, which reflects it’s ability to stimulate an immune response, whether cell-mediated or humoral, against the targeted pathogen. According to computational findings from our study, the candidate vaccine construct demonstrated an immunogenicity score of 0.77 and antigenic probability score of 0.9425, as predicted by the VaxiJen and ANTIGENPro server, respectively. An average antigenic probability score of ≥ 0.8 is typically preferred for a vaccine candidate^85^. Therefore, our obtained score suggests that the newly designed subunit vaccine candidate is likely to exhibit antigenic properties. Moreover, the solubility probability determined by SolPro^86^ was 0.694. The schematic representation of the construct is shown in Fig. 3. The vaccine construct fulfilled all the criteria of a potential vaccine construct, warranting future evaluation.

#### Prediction of physicochemical property

The physicochemical property of the vaccine was analyzed through the ProtParam online server^87^. This analysis provides various information about the protein like molecular weight, theoretical pI of the protein, hydropathic index, aliphatic index etc., The molecular weight of the predicted vaccine is 19 kDa and the theoretical pI was 8.72 which states that the protein is stable. Further, the average of the hydropathic index was −0.609 suggesting the hydrophilic nature of the protein that represents strong interactions with the water molecules. The aliphatic index of the vaccine was 62.75 indicating the stability of the protein in various temperatures. The instability score was 36.76 stating the stability which initiates an immunogenic reaction.

The vaccine construct was analyzed for various PTMs. The analysis conducted using the NetCGlyc-1.0 server indicated the absence of C-glycosylation sites in the vaccine construct. Phosphorylation modification prediction by the NetPhos-3.0 server identified 11 phosphorylation sites (Serine: 5, Threonine: 4, Tyrosine: 2) within the construct. Phosphorylated peptides or epitopes show high affinity towards cytotoxic T lymphocytes and thus play an important role fostering specific and robust immune responses^88^. No N-acetylation site was detected by the NetAcet-1.0 server. Furthermore, there were no lipid PTMs at the N-terminal glycine myristoyl, and GPI-modification, as predicted by the MyrPS/NMT and the big-PI/GPI animals servers, respectively.

**Figure 4:**
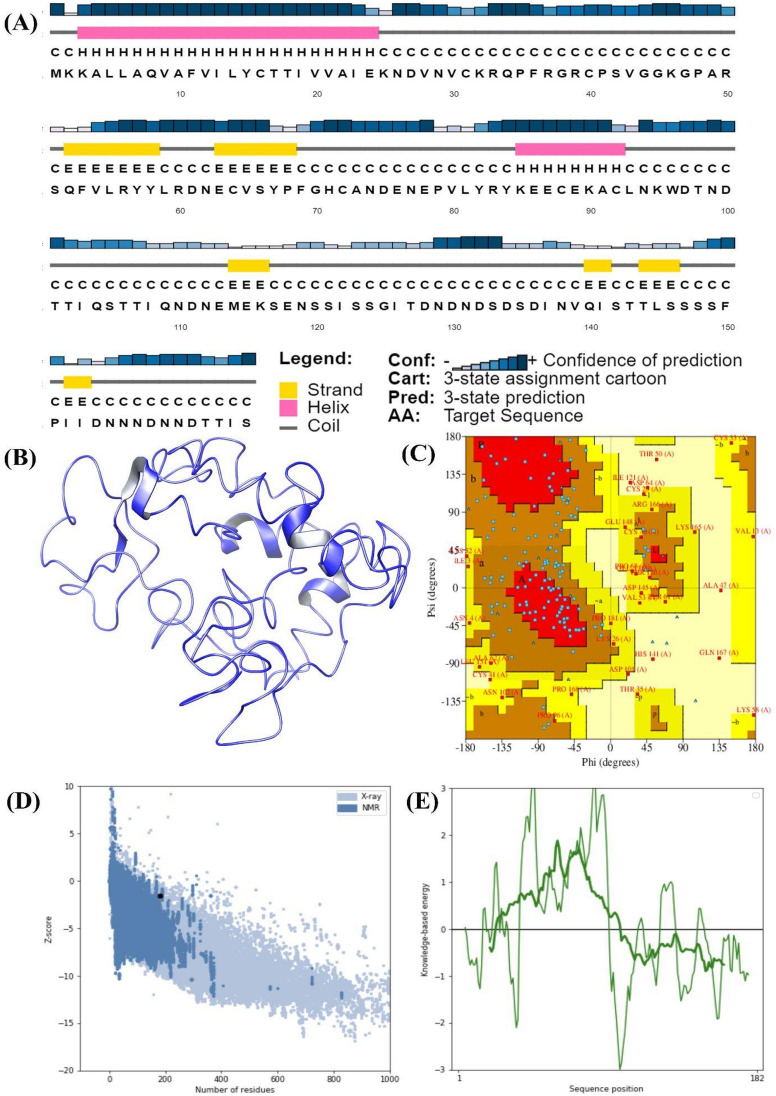
Secondary and tertiary structure of the multi-epitope vaccine construct. (**A**) Secondary structure predicted through PSIPRED, (**B**) modeled structure and (**C**) Ramachandran plot validation of modeled structure, (**D**) ProSA energy score, (**E**) energy profile of the modeled structure.

The secondary structure of the selected vaccine construct was predicted through the PSIPRED online server is presented in Fig. 4A. The structure represented that it possessed alpha helix, beta sheets and coil. The protein model was generated through the online server i-TASSER-MTD which produced five models. Those models were validated through the Ramachandran model (Fig. 4C) and the best model (Fig. 4B) was selected for the docking strategies. The predicted structure along with the validation has been presented in Fig. 4C–E. Further, the ProSA analysis generated the value of −1.6 stating that the structure possesses good quality quantifying the difference between the structure's total energy and an energy distribution produced from random conformations. This analysis is presented in Fig. 4D which plots the energies as a function of amino acid sequence position, demonstrating the local model quality. The energy profile (Fig. 4E) represented that the model acquired stable energy and can be taken further for molecular modeling studies. Further, the validation of the multi epitope fusion vaccines modelled has been presented in Supplementary Fig. S5 stating that the model has acquired stability and can be used further for the molecular modelling studies.

#### Interaction of the constructed vaccine with TLRs and HLA alleles

The modelled vaccine was further analyzed for the interaction with the TLR4, TLR3 human proteins and HLA alleles. Nine HLA alleles that showed binding with the MHC-I epitopes in this study were selected for docking. The interaction studies were performed using HDock. The HDock docking protocol showed a high negative score implying the strong binding energy due to good interaction with human proteins (TLR3 & TLR4 and HLA alleles). The docking scores along with the confidence score of the complexes and the binding affinity of the complexes are given in Table 3.

**Table 3:** Docking scores obtained during the interaction analysis of vaccine with TLRs and HLA alleles.

S. no.	Protein	PDB ID	Docking score	Confidence score	Binding affinity (kcal/mol)
1	TLR3	1ZIW	−318.95	0.9670	−13.7
2	TLR4	3FX1	−351.41	0.9825	−14.2
3	HLA A 01:01	4NQX	−237.59	0.8522	−12.1
4	HLA B 57:01	5VUF	−255.20	0.8913	−13.1
5	HLA A 02:06	3OXR	−245.72	0.8715	−12.7
6	HLA A 02:03	3OX8	−245.65	0.8714	−12.6
7	HLA B 07:02	6AT5	−254.49	0.8899	−12.9
8	HLA A 02:01	7M8S	−256.23	0.8933	−13.1
9	HLA A 68:01	6PBH	−248.79	0.8782	−12.5
10	HLA B 15:01	6UZP	−253.37	0.8877	−12.7
11	HLA A 11:01	5WJL	−240.03	0.8782	−11.6

#### Molecular dynamics simulation of the Apo-vaccine and complex

**Figure 5:**
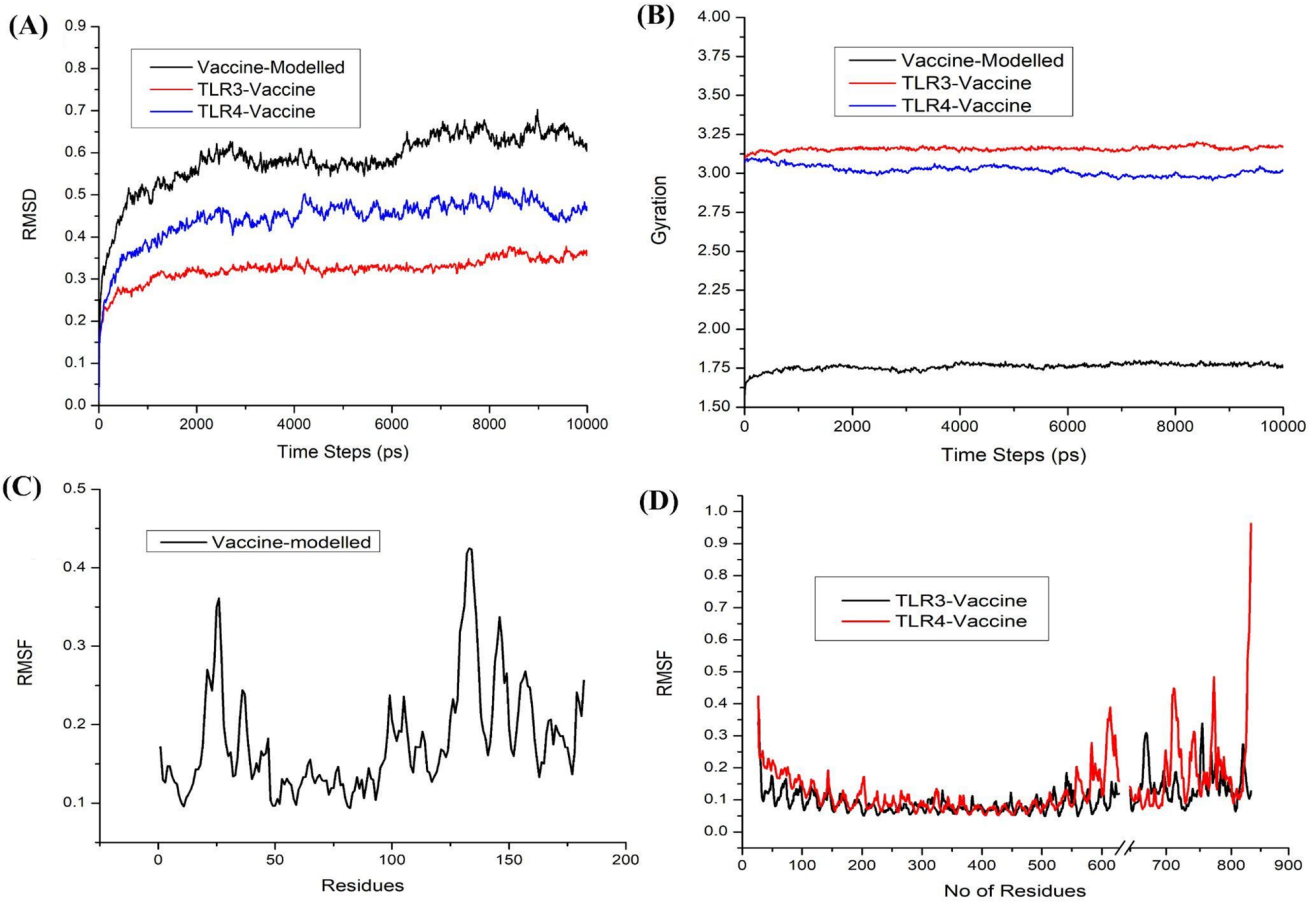
Molecular dynamics simulations of Apo-vaccine and the complex. (**A**) RMSD and (**B**) radius of gyration of the modeled vaccine and docked complex of TLR3-vaccine and TLR4-vaccine. (**C**) RMSF of the modelled vaccine and (**D**) RMSF of the docked complex TLR3-vaccine and TLR4-vaccine.

The stability of the modeled structure along with the protein–protein complexes were subjected to a 10 ns simulation in the explicit water using SiBioLead which is an online biocomputing platform for computational drug discovery which uses GROMACS (Groningen Machine for Chemical Simulations). The modeled structure attained stability after 4 to 6 ns and level of stability fluctuated till 10 ns time period (Fig. 5A). The comparison of complexes and the modeled proteins showed that the complex attained better stability than the modeled structure (Fig. 5A). The gyration graph supported the RMSD of the complex and the vaccine proteins (Fig. 5B). Further, Fig. 5C represented the Root mean square fluctuation of the modeled vaccine target obtained, whereas Fig. 5D represented the RMSF of the bounded complex. There is a break between the residues ranging from 600 to 700 showing the bounded state between the TLRs and the vaccine (Fig. 5D). TLR3 showed minimal fluctuations compared with the TLR4. When compared with the apoprotein, the fluctuations in TLRs-vaccine are minimal, possibly due to the bounded state.

#### Conformational analysis of the HLA allele and the vaccine construct

**Figure 6:**
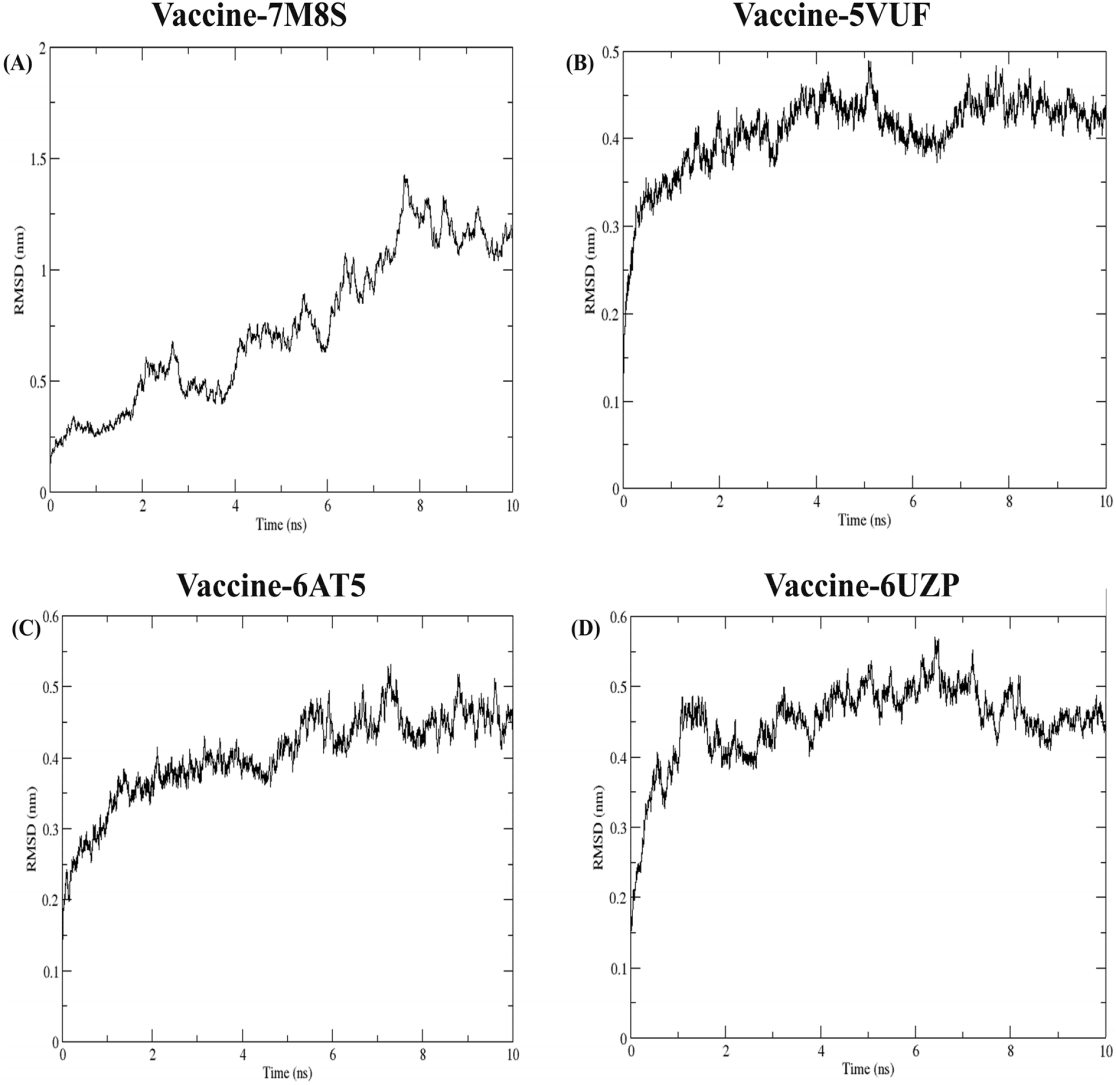
RMSD of the vaccine and the four HLA alleles docked through the HDock web server. (**A**) Vaccine-7M8S complex, (**B**) Vaccine-5VUF complex, (**C**) Vaccine-6AT5, (**D**) Vaccine-6UZP complex.

The HLA alleles bound with the modeled vaccine were retrieved from HDock. A total of nine complexes were selected for the docking strategy (Table 3). Based on the docking and confidence score, four complexes were analyzed further for the molecular dynamics simulation. The protein complex with the respective PDB ID’s 7M8S, 5VUF, 6AT5 and 6UZP showed highest docking scores ranging from −256.23 to −253.37 (Table 3). Whereas the confidence scores varied from 0.89 to 0.8877 (Table 3). While the numbers appear similar, the decimal points were considered in distinguishing the scores. The HLA alleles with the highest confidence scores, also exhibited higher docking scores when compared to the other docked complexes. Therefore, we selected the four HLA alleles that corresponded to the highest confidence scores, and higher docking scores, for subsequent molecular dynamics simulations. The RMSD deviated initially to attain equilibrium at around 8 ns (Fig. 6A). The complex of vaccine and 5VUF, showed stable RMSD after 7 ns around 2.55 nm (Fig. 6B). The same impression was observed in the complex vaccine–6AT5 (Fig. 6C) and vaccine-6UZP (Fig. 6D). Further, the radius of gyration is presented in the supplementary Figure S3 showing the correlation between the RMSD and Radius of gyration. The Hydrogen bond of the complexes presented in the supplementary Fig. S4 indicated the strength of the binding between the vaccine and the HLA alleles throughout the simulation period. Moreover, the RMSD, Radius of Gyration and Hydrogen bond observations were correlated with each other. These analyses strongly suggested a robust interaction between the vaccine and the alleles during the simulation period with the root mean square deviation getting stabilized after the initial equilibrium state. The hydrogen bond interaction showed no loss of hydrogen bonds indicating stable binding. All these features indicated that the HLA alleles when bound with the vaccine exhibited strong stability proving it to be the potential candidate.

#### In silico cloning of vaccine construct

Codon adaptation is a method employed to enhance the expression rates of bacterial genomes by optimizing codons. In the present study, this strategy was implemented to increase the synthesis rate of a vaccine construct in the *E. coli* K12 system, considering the divergent codon usage between humans and the target host. To achieve this, the JCat webserver was used to back-translate and modify the codons of the vaccine construct sequence. Analysis of the modified sequence indicated 52% GC content, which fell within the acceptable range of 30–70%. Additionally, the codon adaptation index was found to be 0.98, suggesting a high potential for the expression of the vaccine construct.

#### Immune simulation of predicted vaccine construct

**Figure 7:**
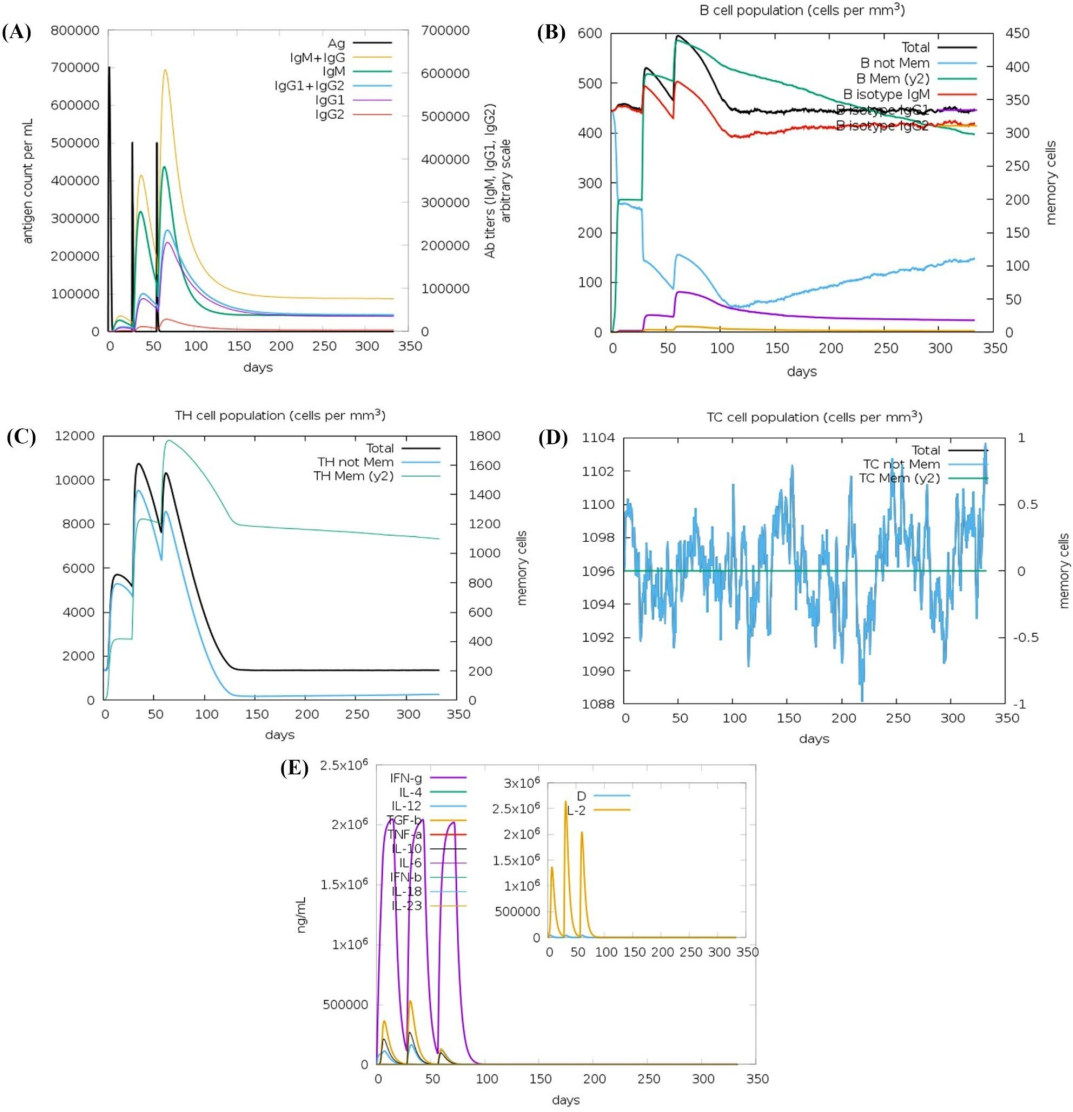
C-ImmSim plots showing the immune response of the multi-epitope vaccine construct. (**A**) The immunoglobulins and their subclasses in response to the injection of the vaccine construct (antigen) at three different time intervals; Antigen inoculations are shown as black vertical lines and immunoglobulin subclasses are indicated as colored peaks, (**B**) B-cell populations following three injections. (**C**) The population of T-helper cells. (**D**) The evolution of CD4 T-helper cell classes during the course of vaccination. (**E**) Levels of cytokine and interleukin after three injections; inset plot shows danger signal and interleukin growth factor IL-2.

The vaccine construct was also analyzed using the C-ImmSim server to simulate the natural immune response in the human body. Upon encountering antigens, the immune reaction is initiated, leading to the production of IgM antibodies, with a smaller proportion of IgG antibodies (Fig. 7A). The production of antibodies is typically low initially and depends on the type of antigenic pathogen. We injected three doses of the vaccine at intervals of three months. By administering the vaccine dosage (antigens) for the first time, the levels of IgM antibodies started to rise (Fig. 7A). Subsequent antigen exposures with different time intervals resulted in a robust immune response characterized by higher levels of IgM and IgG antibodies (Fig. 7A). The levels of IgM, IgG1, and IgG2 antibodies significantly increased, indicating a stronger binding affinity of immunoglobulins for the vaccine construct (antigens) and the establishment of immunological memory. This enhanced immune response leads to improved pathogen elimination upon additional antigenic doses.

The B-cell, CD8+ and CD4+ T-cells exhibited a strong reaction in different cells, accompanied by the formation of memory cells (Fig. 7B–D). The frequency of Helper T cells remained higher throughout the exposure (Fig. 7C). The levels of cytokines during the immune reaction were assessed to determine any potential risks that might lead to complications in the human system, using the Simpson index D graph (Fig. 7E). The low D value indicated that the vaccine construct is safe to use.

For allergenicity assessment of the vaccine, we employed the AlgPred server, which integrates six distinct approaches: (i) IgE mapping, (ii) MEME/Mast motif analysis, (iii, iv) SVM modules based on both amino acid and dipeptide composition, (v) BLAST search against ARPs, and (vi) a hybrid approach combining all parameters. Among these approaches, MEME/Mast motif analysis, BLAST search against ARPs, and IgE mapping consistently indicated that the vaccine construct is non-allergenic. However, SVM modules, using both amino acid and dipeptide compositions, as well as the hybrid approach, suggested allergenicity of the vaccine construct. To ensure a comprehensive evaluation, we further conducted allergenicity analysis using the AllerTop server^89^. The results obtained from this tool corroborated the non-allergenic nature of the vaccine. In light of the combined results from both servers, we concluded that the vaccine candidate is likely non-allergenic, thereby minimizing the risk of allergic reactions during vaccination protocols.

### Analysis of VAH, ALT2, and the fusion of two proteins

To assess the usability of the analysis pipeline for vaccine predictions we tested it with known vaccine targets: VAH (AAD16985), ALT-2 (AAC35355), and the fusion of these two proteins (VAH-ALT-2), created by linking the two proteins with a GGGGS(3) linker. These proteins were selected as multivalent vaccine formulation of *B. malayi* VAH and ALT-2 provided higher protection compared to monovalent formulations of each protein^39^.

Both T- and B-cell epitopes of each protein were identified. Interestingly, the T/B cell epitopes of the fused protein were nearly identical to those of VAH and ALT-2 (Supplementary Table S4). Tertiary structure models were developed for all three proteins, and the rationality of their structures was determined using Ramachandran plots (Supplementary Fig. S5). For ALT-2, where no similar sequences were found in the protein database, i-TASSER-MTD was employed for modeling. In the case of VAH and the fusion protein, similarity was observed with the *B. malayi* venom allergen-like protein-1 (ID: 6ANY) from the Protein Data Bank, leading to the use of MODELLER version 10.4 for modeling^90^. The modeled proteins were then analyzed for interaction with TLR4, TLR3 human proteins, and HLA alleles used for the new vaccine construct. The HDock docking protocol revealed that the fusion protein exhibited the highest negative score in most complexes (Table 4), indicating strong binding energy and favorable interaction with human proteins. The analysis indicated that fusion protein had the combination of the epitopes from both the genes and, importantly, improved binding affinity to human proteins compared to the individual protein. This comprehensive analysis demonstrated the effectiveness of the in-silico methods employed in this study in predicting and validating the proposed vaccine construct.

**Table 4:** Docking scores obtained during the interaction analysis of multi epitope fusion vaccine with TLRs and HLA alleles.

S. no.	Protein	PDB ID	Docking score		
			AAC35355.1	AAD16985	Fusion vaccine
1	TLR3	1ZIW	−320.48	−318.54	−342.80
2	TLR4	3FX1	−315.14	−293.76	−326.63
3	HLA A 01:01	4NQX	−310.29	−291.10	−341.44
4	HLA B 57:01	5VUF	−298.33	−269.47	−316.32
5	HLA A 02:06	3OXR	−301.10	−317.54	−316.32
6	HLA A 02:03	3OX8	−282.33	−305.58	−294.93
7	HLA B 07:02	6AT5	−269.88	−258.83	−297.47
8	HLA A 02:01	7M8S	−280.93	−278.36	−297.88
9	HLA A 68:01	6PBH	−274.22	−312.78	−286.76
10	HLA B 15:01	6UZP	−303.66	−272.48	−316.70
11	HLA A 11:01	5WJL	−295.73	−315.71	−318.68

**Figure 8:**
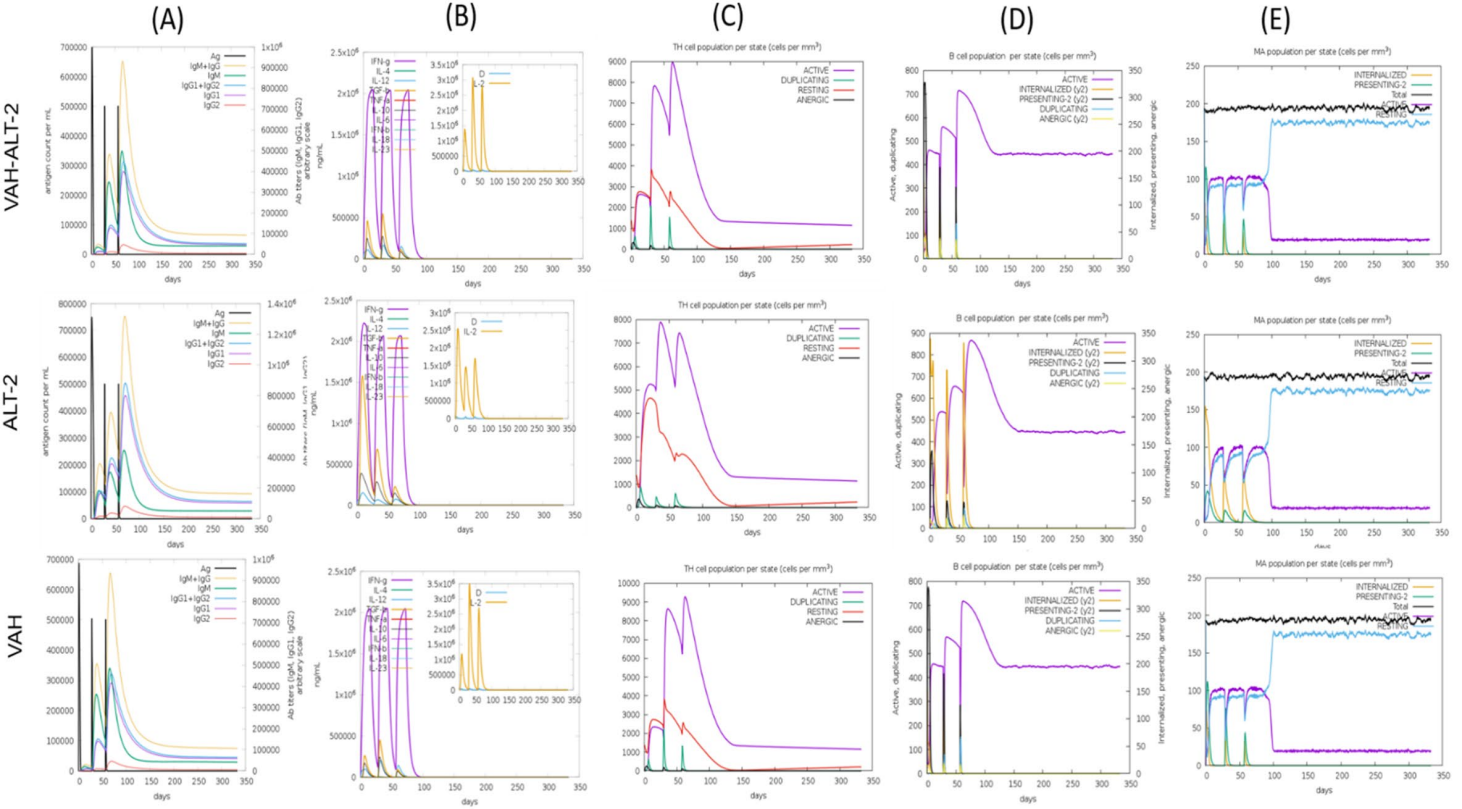
In-silico immune simulation analysis of the candidate vaccine targets, ALT-2, VAH and fused protein (VAH-ALT-2). The simulation was performed for three injections at time steps of 1, 84, and 170. (**A**) Antigen and immunoglobulins (antibodies are sub-divided per isotype); (**B**) concentration of cytokines and interleukins. D in the inset plot is danger signal; (**C**) CD4 T-helper lymphocyte count sub-divided per entity (i.e. active, resting, anergic and duplicating); (**D**) B-lymphocytes population per entity active, presenting on class-II, internalized the antigen, duplicating and anergic; (**E**) macrophage cell population.

Furthermore, all the three proteins were examined for assessing their potential to elicit immune response using C-ImmSim server. The VAH and the VAH-ALT-2 exhibited a comparable immune response pattern, while ALT-2 showed distinct characteristics. Specifically, ALT-2 displayed a decreased level of IgM compared to the other two proteins (Fig. 8A). The secondary and tertiary responses (IgG + IgM, and IgG1 + IgG2 antibodies) were more robust than the primary response for ALT-2 (Fig. 8A). Despite the enhanced secondary and tertiary responses in ALT-2, concerns arose as Interleukin-2 (IL-2) levels were observed to decrease (Fig. 8B). IL-2 is a critical signaling molecule in the immune system, important for the activation and proliferation of CD4+ T helper cells^91^. These cells are involved in coordinating immune responses, providing assistance to other immune cells, and facilitating the differentiation of B cells into antibody-producing plasma cells. Additionally, increased Transforming Growth Factor-beta (TGF-β) levels were noted in ALT-2 (Fig. 8B). TGF-β plays a dual role exhibiting both immunosuppressive and immune-regulatory effects in vaccines and the immune response^92^. It is recognized to inhibit the proliferation of T cells, including CD4+ and CD8+ T cells. The observed rise in TNF-β suggests a complex interplay of factors in the immune response to ALT-2. Furthermore, ALT-2 exhibited a lesser degree of cell duplication of CD4+ T regulatory cells compared to the other two proteins (Fig. 8C). Moreover, the level of antigen internalization by macrophages and by the B-cell lymphocytes showed a spike in ALT-2, but the lower level of antigen presentation was observed (Fig. 8D, E). In summary, while ALT-2 demonstrated favorable secondary and tertiary immune responses, the simultaneous decrease in IL-2 levels and alterations in regulatory T cell populations raises questions about the regulatory mechanisms associated with ALT-2, potentially influencing the balance of immune responses. These findings indicate the potential superiority of the fused protein over ALT-2. However, VAH showed a similar response pattern as observed in the fused protein based on this analysis.

## Conclusion

In conclusion, our study sheds light on the promising role of in silico approaches in the identification of vaccine candidates for the management of filariasis. The in-silico analysis with the goal to identify therapeutic targets offers several advantages. Firstly, it reduces the time required to identify potential targets for medications and vaccines, allowing for a more efficient development process with fewer trials. Additionally, it helps in reducing costs associated with experimental testing. While we acknowledge the unfortunate limitation of any in silico approach, which inherently relies on predictions, the significance of this research lies in its potential to advance our understanding of the disease and provide valuable insights for further experimental studies. The candidates we have identified through computational analysis represent a crucial starting point in the quest for an effective vaccine against filariasis. It is essential to emphasize that the transition from in-silico predictions to real-world vaccine development is a complex and multifaceted process that demands rigorous experimental validation. However, the candidate identified here provide a valuable foundation upon which future research and vaccine development efforts can be built. Future research should focus on extensive experimental studies, immunogenicity assessments in diverse populations, optimization of vaccine formulations, and long-term efficacy evaluations. Additionally, collaborative efforts, epidemiological studies, and exploration of combination strategies will further enhance the prospects of developing a successful filariasis vaccine. However, this holistic approach ensures that the findings from in-silico predictions contribute substantively to the advancement of developing effective preventive measures against filariasis.

### Supplementary Information

Supplementary Information.

## Data Availability

The datasets generated and/or analyzed during the current study are included in the text.
