# Supplementary material for: Identification and prioritisation of potential vaccine candidates using subtractive proteomics and designing of a multi-epitope vaccine against Wuchereria bancrofti
Source: Sci Rep. 2024 Jan 23;14:1970. doi: 10.1038/s41598-024-52457-x (PMC10806236; doi:10.1038/s41598-024-52457-x)
Supplement: Supplementary file 1 — Supplementary Information. [file 41598_2024_52457_MOESM1_ESM.docx]

**Supplementary Information**

**Identification and** **prioritisation of potential vaccine candidates using subtractive proteomics and designing of a multi-epitope vaccine against *Wuchereria bancrofti***

Murali Aarthy^1^, G Navaneetha Pandiyan^1^, R Paramasivan^1^, Ashwani Kumar^2,3^ and Bhavna Gupta^1*^

**Affiliations :**

1 ICMR-Vector Control Research Centre (VCRC), Field Station Madurai-02, TN, India

2 ICMR-Vector Control Research Centre (VCRC), Puducherry, India

3 Present address: Saveetha University, Tandhalam, Chennai-602105, Tamil Nadu, India

**Corresponding author:**

Bhavna Gupta

ICMR-Vector Control Research Centre (VCRC), Field Station Madurai-02, TN , India

bhavnaguptaster@gmail.com; +91 7036123756

**Table S1: List of first 100 BLAST hits with the *Wucherria bancrofti* protein VDM15541.1**

| S.No | Description | Scientific Name | Max Score | Total Score | Query Cover | E value | Per. ident | Acc. Len | Accession |
| --- | --- | --- | --- | --- | --- | --- | --- | --- | --- |
|  | unnamed protein product [Acanthocheilonema viteae] | *Acanthocheilonema viteae* | 174 | 174 | 86% | 2.00E-46 | 60.93 | 3744 | VBB27387.1 |
|  | hypothetical protein ANCCAN_08760 [Ancylostoma caninum] | *Ancylostoma caninum* | 124 | 124 | 41% | 8.00E-31 | 73.91 | 307 | RCN45260.1 |
|  | Kunitz/Bovine pancreatic trypsin inhibitor domain protein [Ancylostoma ceylanicum] | *Ancylostoma ceylanicum* | 123 | 123 | 41% | 8.00E-29 | 73.91 | 3600 | EPB71358.1 |
|  | hypothetical protein Y032_0833g2587 [Ancylostoma ceylanicum] | *Ancylostoma ceylanicum* | 119 | 119 | 41% | 2.00E-28 | 73.91 | 399 | EYC37031.1 |
|  | hypothetical protein ANCDUO_10416 [Ancylostoma duodenale] | *Ancylostoma duodenale* | 119 | 119 | 43% | 6.00E-31 | 71.83 | 132 | KIH59354.1 |
|  | hypothetical protein ANCDUO_15944 [Ancylostoma duodenale] | *Ancylostoma duodenale* | 79 | 79 | 28% | 7.00E-16 | 70.21 | 75 | KIH53913.1 |
|  | hypothetical protein Angca_000848 [Angiostrongylus cantonensis] | *Angiostrongylus cantonensis* | 120 | 120 | 40% | 1.00E-31 | 77.61 | 90 | KAE9413185.1 |
|  | unnamed protein product [Angiostrongylus costaricensis] | *Angiostrongylus costaricensis* | 122 | 122 | 40% | 8.00E-29 | 77.61 | 590 | VDM63339.1 |
|  | Papilin [Aphelenchoides besseyi] | *Aphelenchoides besseyi* | 124 | 124 | 49% | 1.00E-29 | 65.85 | 507 | KAI6186676.1 |
|  | Thyroglobulin type-1 and Proteinase inhibitor I2 domain containing protein [Aphelenchoides besseyi] | *Aphelenchoides besseyi* | 119 | 119 | 42% | 2.00E-27 | 68.57 | 2327 | KAI6199887.1 |
|  | Thyroglobulin type-1 and Proteinase inhibitor I2 domain containing protein [Aphelenchoides fujianensis] | *Aphelenchoides fujianensis* | 120 | 120 | 40% | 1.00E-27 | 76.12 | 2387 | KAI6228908.1 |
|  | unnamed protein product [Auanema sp. JU1783] | *Auanema sp. JU1783* | 120 | 120 | 43% | 8.00E-28 | 70.83 | 3119 | CAI4223013.1 |
|  | Kunitz/Bovine pancreatic trypsin inhibitor domain containing protein [Brugia malayi] | *Brugia malayi* | 239 | 239 | 92% | 1.00E-69 | 80.13 | 3040 | XP_042938220.1 |
|  | BMA-MLT-11, isoform b [Brugia malayi] | *Brugia malayi* | 239 | 239 | 92% | 1.00E-69 | 80.13 | 3040 | CDP90581.1 |
|  | BMA-MLT-11, isoform a [Brugia malayi] | *Brugia malayi* | 238 | 238 | 92% | 4.00E-69 | 80.13 | 2425 | CDP90580.1 |
|  | BMA-MLT-11, isoform d [Brugia malayi] | *Brugia malayi* | 237 | 237 | 92% | 1.00E-68 | 80.13 | 1971 | CDP90582.1 |
|  | Bm13191 [Brugia malayi] | *Brugia malayi* | 176 | 176 | 55% | 4.00E-54 | 89.01 | 89 | CDQ08439.1 |
|  | unnamed protein product [Brugia pahangi] | *Brugia pahangi* | 234 | 234 | 92% | 1.00E-67 | 82.69 | 3013 | VDN92962.1 |
|  | unnamed protein product [Brugia timori] | *Brugia timori* | 202 | 202 | 78% | 7.00E-57 | 79.07 | 953 | VDO34786.1 |
|  | unnamed protein product [Bursaphelenchus okinawaensis] | *Bursaphelenchus okinawaensis* | 127 | 127 | 60% | 3.00E-30 | 52.48 | 2460 | CAD5225297.1 |
|  | unnamed protein product [Bursaphelenchus xylophilus] | *Bursaphelenchus xylophilus* | 124 | 124 | 60% | 3.00E-29 | 53.92 | 2251 | CAD5231496.1 |
|  | unnamed protein product [Caenorhabditis angaria] | *Caenorhabditis angaria* | 126 | 126 | 57% | 7.00E-30 | 61.05 | 2784 | CAI5451245.1 |
|  | unnamed protein product [Caenorhabditis auriculariae] | *Caenorhabditis auriculariae* | 122 | 122 | 43% | 1.00E-31 | 72.22 | 177 | CAD6193630.1 |
|  | unnamed protein product [Caenorhabditis bovis] | *Caenorhabditis bovis* | 125 | 125 | 53% | 1.00E-29 | 59.09 | 3024 | CAB3399020.1 |
|  | hypothetical protein CAEBREN_18581 [Caenorhabditis brenneri] | *Caenorhabditis brenneri* | 122 | 122 | 70% | 2.00E-28 | 53.85 | 1249 | EGT33025.1 |
|  | hypothetical protein L5515_009906 [Caenorhabditis briggsae] | *Caenorhabditis briggsae* | 122 | 122 | 48% | 2.00E-28 | 67.5 | 3019 | UMM38522.1 |
|  | hypothetical protein L5515_009906 [Caenorhabditis briggsae] | *Caenorhabditis briggsae* | 122 | 122 | 48% | 2.00E-28 | 67.5 | 3036 | UMM38524.1 |
|  | hypothetical protein L3Y34_010106 [Caenorhabditis briggsae] | *Caenorhabditis briggsae* | 122 | 122 | 48% | 2.00E-28 | 67.5 | 3036 | ULT92782.1 |
|  | hypothetical protein L5515_009906 [Caenorhabditis briggsae] | *Caenorhabditis briggsae* | 122 | 122 | 48% | 2.00E-28 | 67.5 | 3010 | UMM38523.1 |
|  | hypothetical protein L3Y34_010106 [Caenorhabditis briggsae] | *Caenorhabditis briggsae* | 122 | 122 | 48% | 2.00E-28 | 67.5 | 3027 | ULT92781.1 |
|  | Papilin [Caenorhabditis elegans] | *Caenorhabditis elegans* | 122 | 122 | 63% | 1.00E-28 | 55.77 | 3129 | NP_001256938.1 |
|  | Papilin [Caenorhabditis elegans] | *Caenorhabditis elegans* | 122 | 122 | 63% | 1.00E-28 | 55.77 | 3120 | NP_001256941.1 |
|  | Papilin [Caenorhabditis elegans] | *Caenorhabditis elegans* | 122 | 122 | 63% | 2.00E-28 | 55.77 | 2603 | NP_001256939.1 |
|  | Papilin [Caenorhabditis elegans] | *Caenorhabditis elegans* | 122 | 122 | 48% | 2.00E-28 | 67.5 | 3000 | NP_001256942.1 |
|  | Papilin [Caenorhabditis elegans] | *Caenorhabditis elegans* | 121 | 121 | 48% | 2.00E-28 | 67.5 | 2373 | NP_001256945.1 |
|  | Papilin [Caenorhabditis elegans] | *Caenorhabditis elegans* | 121 | 121 | 48% | 3.00E-28 | 67.5 | 2382 | NP_001256944.1 |
|  | Papilin [Caenorhabditis elegans] | *Caenorhabditis elegans* | 121 | 121 | 63% | 3.00E-28 | 55.77 | 2175 | NP_001256940.1 |
|  | Papilin [Caenorhabditis elegans] | *Caenorhabditis elegans* | 121 | 121 | 48% | 3.00E-28 | 67.5 | 2527 | NP_001256943.1 |
|  | hypothetical protein B9Z55_022016 [Caenorhabditis nigoni] | *Caenorhabditis nigoni* | 122 | 122 | 48% | 2.00E-28 | 67.5 | 3020 | PIC30935.1 |
|  | CRE-MLT-11 protein [Caenorhabditis remanei] | *Caenorhabditis remanei* | 122 | 122 | 55% | 2.00E-28 | 61.54 | 3019 | EFP00691.1 |
|  | unnamed protein product [Caenorhabditis sp. 36 PRJEB53466] | *Caenorhabditis sp. 36 PRJEB53466* | 121 | 121 | 55% | 4.00E-28 | 60.44 | 3153 | CAI2352529.1 |
|  | unnamed protein product [Cercopithifilaria johnstoni] | *Cercopithifilaria johnstoni* | 180 | 180 | 93% | 9.00E-49 | 60 | 1579 | CAG9537700.1 |
|  | Kunitz/Bovine pancreatic trypsin inhibitor domain protein [Dictyocaulus viviparus] | *Dictyocaulus viviparus* | 124 | 124 | 52% | 2.00E-29 | 61.63 | 906 | KJH47398.1 |
|  | hypothetical protein WR25_11985 [Diploscapter pachys] | *Diploscapter pachys* | 125 | 125 | 53% | 2.00E-29 | 60 | 3210 | PAV69501.1 |
|  | hypothetical protein WR25_26744 [Diploscapter pachys] | *Diploscapter pachys* | 123 | 123 | 50% | 6.00E-29 | 63.86 | 3009 | PAV62906.1 |
|  | Papilin [Dirofilaria immitis] | *Dirofilaria immitis* | 160 | 160 | 86% | 1.00E-41 | 56.34 | 2818 | MCP9264506.1 |
|  | kunitz/Bovine pancreatic trypsin inhibitor domain-containing protein [Ditylenchus destructor] | *Ditylenchus destructor* | 122 | 122 | 60% | 2.00E-28 | 55.88 | 2922 | KAI1722806.1 |
|  | unnamed protein product [Dracunculus medinensis] | *Dracunculus medinensis* | 120 | 120 | 40% | 8.00E-28 | 77.61 | 1481 | VDN52924.1 |
|  | unnamed protein product [Enterobius vermicularis] | *Enterobius vermicularis* | 120 | 120 | 44% | 4.00E-28 | 71.62 | 1054 | VDD92168.1 |
|  | NAD(P)H-quinone oxidoreductase subunit 5, chloroplastic [Globodera pallida] | *Globodera pallida* | 109 | 109 | 41% | 5.00E-24 | 70.59 | 1512 | KAI3410164.1 |
|  | unnamed protein product [Gongylonema pulchrum] | *Gongylonema pulchrum* | 129 | 129 | 46% | 7.00E-34 | 75.32 | 205 | VDK31090.1 |
|  | Thyroglobulin type-1 and Proteinase inhibitor I2 domain containing protein [Haemonchus contortus] | *Haemonchus contortus* | 123 | 123 | 43% | 8.00E-29 | 72.22 | 2067 | CDJ84543.1 |
|  | unnamed protein product [Haemonchus placei] | *Haemonchus placei* | 120 | 120 | 40% | 9.00E-28 | 76.12 | 1598 | VDO28023.1 |
|  | hypothetical protein FO519_001163 [Halicephalobus sp. NKZ332] | *Halicephalobus sp. NKZ332* | 117 | 117 | 46% | 9.00E-27 | 67.95 | 2430 | KAE9555585.1 |
|  | unnamed protein product [Heligmosomoides polygyrus] | *Heligmosomoides polygyrus* | 120 | 120 | 41% | 8.00E-29 | 73.91 | 382 | VDP01904.1 |
|  | unnamed protein product [Litomosoides sigmodontis] | *Litomosoides sigmodontis* | 172 | 172 | 95% | 7.00E-46 | 55.97 | 3651 | VDK68293.1 |
|  | CBR-MLT-11 protein [Loa loa] | *Loa loa* | 181 | 181 | 79% | 2.00E-49 | 67.18 | 1014 | XP_020305261.1 |
|  | unnamed protein product [Meloidogyne enterolobii] | *Meloidogyne enterolobii* | 105 | 105 | 39% | 9.00E-23 | 67.69 | 1701 | CAD2177646.1 |
|  | unnamed protein product [Meloidogyne enterolobii] | *Meloidogyne enterolobii* | 105 | 105 | 39% | 9.00E-23 | 67.69 | 1910 | CAD2164986.1 |
|  | unnamed protein product [Meloidogyne enterolobii] | *Meloidogyne enterolobii* | 105 | 105 | 41% | 1.00E-22 | 64.71 | 1018 | CAD2194969.1 |
|  | unnamed protein product [Meloidogyne enterolobii] | *Meloidogyne enterolobii* | 84.7 | 84.7 | 26% | 2.00E-16 | 79.07 | 238 | CAD2206339.1 |
|  | unnamed protein product [Meloidogyne enterolobii] | *Meloidogyne enterolobii* | 85.9 | 85.9 | 26% | 9.00E-16 | 79.07 | 2440 | CAD2178599.1 |
|  | hypothetical protein NECAME_06859 [Necator americanus] | *Necator americanus* | 115 | 115 | 50% | 8.00E-30 | 64.29 | 89 | XP_013306765.1 |
|  | unnamed protein product [Onchocerca flexuosa] | *Onchocerca flexuosa* | 136 | 136 | 89% | 1.00E-35 | 48.75 | 300 | VDP18809.1 |
|  | hypothetical protein X798_05774 [Onchocerca flexuosa] | *Onchocerca flexuosa* | 139 | 139 | 68% | 1.00E-34 | 55.56 | 2780 | OZC07196.1 |
|  | unnamed protein product [Onchocerca ochengi] | *Onchocerca ochengi* | 167 | 167 | 89% | 4.00E-44 | 59.86 | 2883 | VDK62881.1 |
|  | NAD(P)H-quinone oxidoreductase subunit 5, chloroplastic [Parelaphostrongylus tenuis] | *Parelaphostrongylus tenuis* | 123 | 123 | 46% | 5.00E-33 | 70 | 106 | KAJ1345671.1 |
|  | mlt-11 [Pristionchus pacificus] | *Pristionchus pacificus* | 111 | 111 | 40% | 8.00E-25 | 70.15 | 3742 | KAF8386888.1 |
|  | unnamed protein product [Soboliphyme baturini] | *Soboliphyme baturini* | 80.9 | 80.9 | 50% | 4.00E-14 | 44.58 | 468 | VDP03745.1 |
|  | hypothetical protein L596_011346 [Steinernema carpocapsae] | *Steinernema carpocapsae* | 120 | 120 | 43% | 3.00E-28 | 72.22 | 521 | TKR86833.1 |
|  | thyroglobulin type-1 repeat-containing domain protein [Teladorsagia circumcincta] | *Teladorsagia circumcincta* | 119 | 119 | 41% | 2.00E-28 | 71.01 | 406 | PIO71742.1 |
|  | unnamed protein product [Thelazia callipaeda] | *Thelazia callipaeda* | 129 | 129 | 52% | 6.00E-33 | 65.52 | 292 | VDM99409.1 |
|  | Papilin [Toxocara canis] | *Toxocara canis* | 120 | 120 | 40% | 8.00E-28 | 77.61 | 779 | KHN73800.1 |
|  | unnamed protein product [Toxocara canis] | *Toxocara canis* | 91.7 | 91.7 | 26% | 8.00E-18 | 86.36 | 1067 | VDM37650.1 |
|  | Papilin [Trichinella britovi] | *Trichinella britovi* | 79.3 | 79.3 | 47% | 1.00E-13 | 41.03 | 1782 | KRY58323.1 |
|  | Papilin [Trichinella britovi] | *Trichinella britovi* | 79.3 | 79.3 | 47% | 2.00E-13 | 41.03 | 1741 | KRY58324.1 |
|  | Papilin [Trichinella britovi] | *Trichinella britovi* | 79.3 | 79.3 | 47% | 2.00E-13 | 41.03 | 1766 | KRY58326.1 |
|  | Papilin [Trichinella britovi] | *Trichinella britovi* | 79.3 | 79.3 | 47% | 2.00E-13 | 41.03 | 1767 | KRY58325.1 |
|  | Papilin [Trichinella nativa] | *Trichinella nativa* | 79.7 | 79.7 | 47% | 1.00E-13 | 41.03 | 1730 | KRZ50100.1 |
|  | Papilin [Trichinella nativa] | *Trichinella nativa* | 79.7 | 79.7 | 47% | 1.00E-13 | 41.03 | 1705 | KRZ50101.1 |
|  | Papilin [Trichinella nativa] | *Trichinella nativa* | 79.7 | 79.7 | 47% | 1.00E-13 | 41.03 | 1746 | KRZ50099.1 |
|  | Papilin [Trichinella nelsoni] | *Trichinella nelsoni* | 79.7 | 79.7 | 47% | 1.00E-13 | 41.03 | 1690 | KRX26930.1 |
|  | Papilin [Trichinella nelsoni] | *Trichinella nelsoni* | 79.7 | 79.7 | 47% | 1.00E-13 | 41.03 | 1695 | KRX26934.1 |
|  | Papilin [Trichinella nelsoni] | *Trichinella nelsoni* | 79.7 | 79.7 | 47% | 1.00E-13 | 41.03 | 1659 | KRX26931.1 |
|  | Papilin [Trichinella nelsoni] | *Trichinella nelsoni* | 79.7 | 79.7 | 47% | 1.00E-13 | 41.03 | 1676 | KRX26933.1 |
|  | Papilin [Trichinella nelsoni] | *Trichinella nelsoni* | 79.7 | 79.7 | 47% | 1.00E-13 | 41.03 | 1613 | KRX26932.1 |
|  | Papilin [Trichinella patagoniensis] | *Trichinella patagoniensis* | 80.1 | 80.1 | 47% | 1.00E-13 | 41.03 | 1840 | KRY22115.1 |
|  | Papilin [Trichinella patagoniensis] | *Trichinella patagoniensis* | 80.1 | 80.1 | 47% | 1.00E-13 | 41.03 | 1854 | KRY22117.1 |
|  | Papilin [Trichinella patagoniensis] | *Trichinella patagoniensis* | 79.7 | 79.7 | 47% | 1.00E-13 | 41.03 | 1750 | KRY22116.1 |
|  | Papilin [Trichinella pseudospiralis] | *Trichinella pseudospiralis* | 81.6 | 81.6 | 53% | 3.00E-14 | 41.57 | 1615 | KRX89767.1 |
|  | Papilin [Trichinella pseudospiralis] | *Trichinella pseudospiralis* | 81.6 | 81.6 | 53% | 3.00E-14 | 41.57 | 1611 | KRY87127.1 |
|  | Papilin [Trichinella pseudospiralis] | *Trichinella pseudospiralis* | 81.6 | 81.6 | 53% | 3.00E-14 | 41.57 | 1612 | KRZ21476.1 |
|  | Papilin [Trichinella sp. T6] | *Trichinella sp. T6* | 79.7 | 79.7 | 47% | 1.00E-13 | 41.03 | 1746 | KRX73085.1 |
|  | Papilin [Trichinella sp. T6] | *Trichinella sp. T6* | 79.7 | 79.7 | 47% | 1.00E-13 | 41.03 | 1730 | KRX73086.1 |
|  | hypothetical protein D918_03308 [Trichuris suis] | *Trichuris suis* | 83.2 | 83.2 | 75% | 8.00E-15 | 40 | 3115 | KHJ46260.1 |
|  | hypothetical protein M513_08035 [Trichuris suis] | *Trichuris suis* | 81.6 | 81.6 | 75% | 2.00E-14 | 40 | 1417 | KFD51135.1 |
|  | Putative kunitz [Trichuris trichiura] | *Trichuris trichiura* | 80.1 | 80.1 | 46% | 1.00E-13 | 48.05 | 1182 | CDW52091.1 |
|  | unnamed protein product [Wuchereria bancrofti] | *Wuchereria bancrofti* | 340 | 340 | 100% | 6.00E-118 | 100 | 165 | VDM15541.1 |
|  | unnamed protein product [Wuchereria bancrofti] | *Wuchereria bancrofti* | 167 | 167 | 51% | 4.00E-50 | 98.82 | 106 | VDM17902.1 |
|  | hypothetical protein WUBG_17936 [Wuchereria bancrofti] | *Wuchereria bancrofti* | 98.6 | 98.6 | 31% | 1.00E-23 | 100 | 71 | EJW71158.1 |

Table S2:

| **S.No** | **NetCTL epitopes** | **MHC-I binding epitopes** | **Immunogenic Epitopes** |
| --- | --- | --- | --- |
|  | AQVAFVILY(1.1972) | KALLAQVAF (0.1780) | TTIVVAIEK (-0.6825) |
|  | ARSQFVLRY (0.9857 | MEKSENSSI (0.9078) | AQVAFVILY 1.1972 |
|  | RSQFVLRYY (1.0745 | RSQFVLRYY (1.0745) | RSQFVLRYY 1.0745 |
|  | LRDNECVSY (0.4116 | SQFVLRYYL (1.5134) | ENEPVLYRY 0.4549 |
|  | STTLSSSSF (0.6372 | AQVAFVILY (1.1972) | ALLAQVAFV 0.3024 |
|  | ALLAQVAFV (0.3024 | STTLSSSSF (0.6372) | ARSQFVLRY 0.9857 |
|  | LLAQVAFVI (0.5700 | TTIQNDNEM (0.1205) |  |
|  | FVILYCTTI (1.3566 | ENEPVLYRY  (0.4549) |  |
|  | VILYCTTIV (-0.0210 | TTIVVAIEK (-0.6825) |  |
|  | ILYCTTIVV (-0.2948) | ILYCTTIVV (-0.2948) |  |
|  | SQFVLRYYL (1.5134 | KEECEKACL (1.0372) |  |
|  | TLSSSSFPI (0.6044) ) | GPARSQFVL (0.3563) |  |
|  | TTIVVAIEK -(0.6825) | LAQVAFVIL (0.8483) |  |
|  | YYLRDNECV (0.7449) | KWDTNDTTI (0.5707) |  |
|  | KWDTNDTTI (0.5707) | TLSSSSFPI (0.6044) |  |
|  | ENEPVLYRY (0.4549) | LRDNECVSY (0.4116) |  |
|  | TTIQNDNEM (0.1205) | YYLRDNECV (0.7449) |  |
|  | GPARSQFVL (0.3563 ) | ARSQFVLRY (0.9857) |  |
|  | KALLAQVAF (0.1780) | LLAQVAFVI (0.5700) |  |
|  | LAQVAFVIL (0.8483) | ANDENEPVL (0.1003) |  |
|  | GRCPSVGGK (2.1941) | FVILYCTTI (2.1941) |  |
|  | YRYKEECEK (1.5245) | ALLAQVAFV (0.3024) |  |
|  | ANDENEPVL (0.1003) | VILYCTTIV (-0.0210) |  |
|  | KEECEKACL (1.0372) | GRCPSVGGK (2.1941) |  |
|  | MEKSENSSI (0.9078) | YRYKEECEK (1.5245 ) |  |

**Table S3: B cell epitopes VDM15541.1 identified by three different methods**

| **S.No** | **IDEB** | **ABCpred** | **BCPred** |
| --- | --- | --- | --- |
|  | E | STTIQNDNEMEKSENS | GKGPARSQ |
|  | CVSY | VVAIEKNDVNVCKRQP | RDNECVS |
|  | FGHCANDENEPVLYR | PARSQFVLRYYLRDNE | GHCANDENEPV |
|  | NDVNVCKRQPFRGRCPSVGGKGPA | GHCANDENEPVLYRYK | RYKEECEKAC |
|  | LNKWDTNDTTIQSTTIQNDNEMEKSENSS | KEECEKACLNKWDTND | NKWDTNDTTIQSTTIQNDNEMEKSENSSISSGITDNDNDSDSDIN |
|  | ITDNDNDSDSD | DVNVCKRQPFRGRCPS | IDNNNDNNDTTIS |
|  | STTLSSSSFPIIDNNNDNNDT | NECVSYPFGHCANDEN |  |
|  |  | PFRGRCPSVGGKGPAR |  |
|  |  | PVLYRYKEECEKACLN |  |
|  |  | LSSSSFPIIDNNNDNN |  |
|  |  | KKALLAQVAFVILYCT |  |
|  |  | ITDNDNDSDSDINVQI |  |

**Table S4: Selected epitopes of VAH, ALT-2, and VAH-ALT-2 fusion protein**

| **S.No** |  | **VAH** | **ALT-2** | **VAH-ALT-2 (fusion protein)** |
| --- | --- | --- | --- | --- |
|  | MHC-1 | GIGENVYAY |  | GIGENVYAY |
|  | MHC-2 | ELYKHNPSNNLTDDV | DEYVTKGEVVETDGK  GDEYVTKGEVVETDG | ELYKHNPSNNLTDDV  DEYVTKGEVVETDGK  GDEYVTKGEVVETDG |
|  | B-cell | GQLTPQQR | ASESDEEFDDGSNDETDDKEDEGNSEGGDEYVTKGE  VVETDGKKKECSSHEACYDQREPQAWCRPNENQSWTDKGCFCED | ASESDEEFDDGSNDETDDKEDEGNSEGGDEYVTKGEVVETDGKKKECSSHEACYDQREPQAWCRPNENQSWTDKGCFCED |
|  |  | LLIRGKLKNRN |  | GQLTPQQR |
|  |  | GEPCKKDGDCNTKKCA |  | LLIRGKLKNRN |
|  |  | QCVFGHSPRNQR |  | QCVFGHSPRNQR |
|  |  | VRQNNKF |  | CQLENSA |
|  |  | CQLENSA |  | TAGTEAG |
|  |  | ELYKHNP |  | GEPCKKDGDCNTKKCA |
|  |  | TAGTEAG |  | LPELYKHNPSNNLTDD |
|  |  |  |  | ELYKHNP |
|  |  |  |  | GGGGSGGGGSGGGGSM |

**Supplementary Figures**


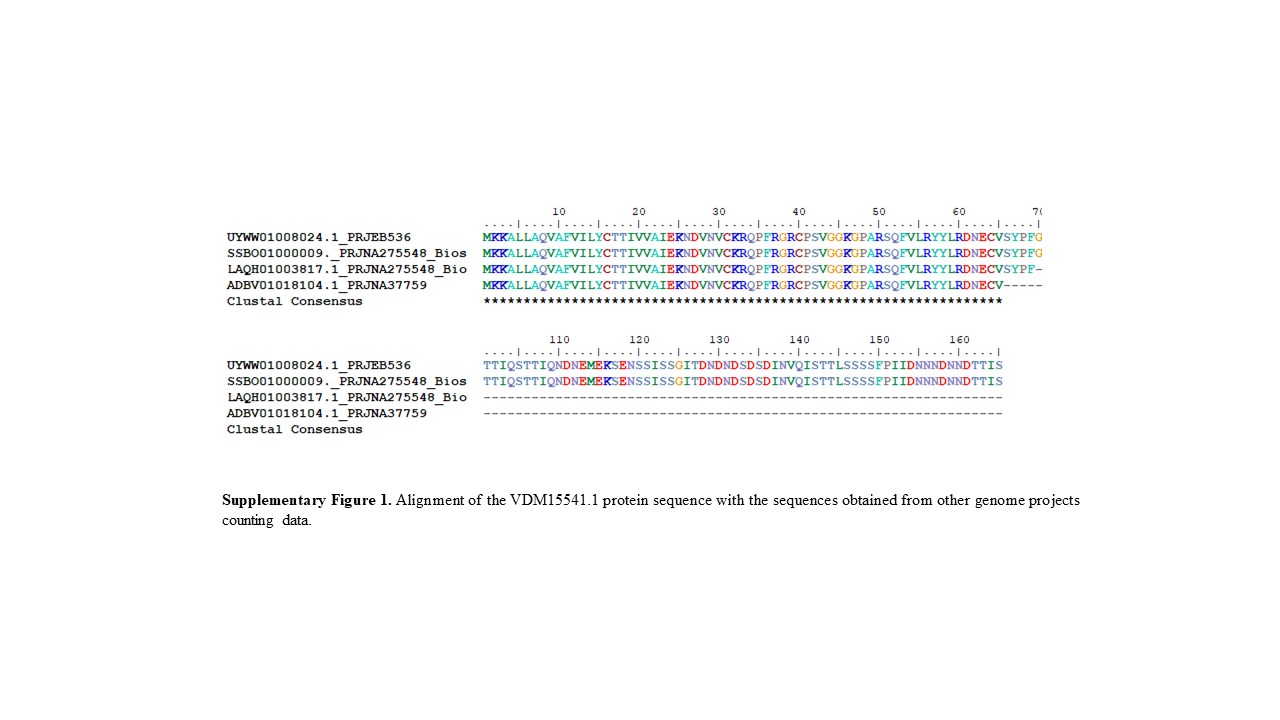


**Supplementary Figure S1: Alignment of the VDM15541.1 protein sequence with the sequences obtained from other genome projects counting data.**


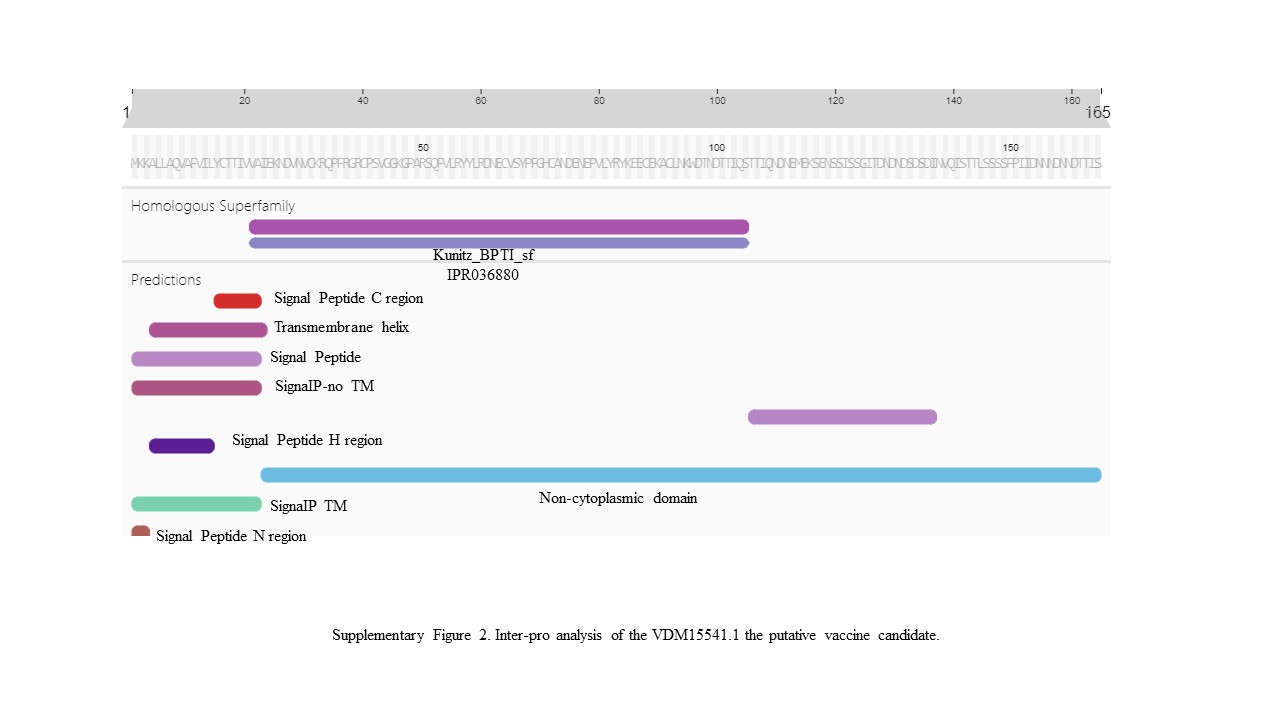


**Supplementary Figure S2: Inter-pro analysis of the VDM15541.1 the putative vaccine candidate.**

**
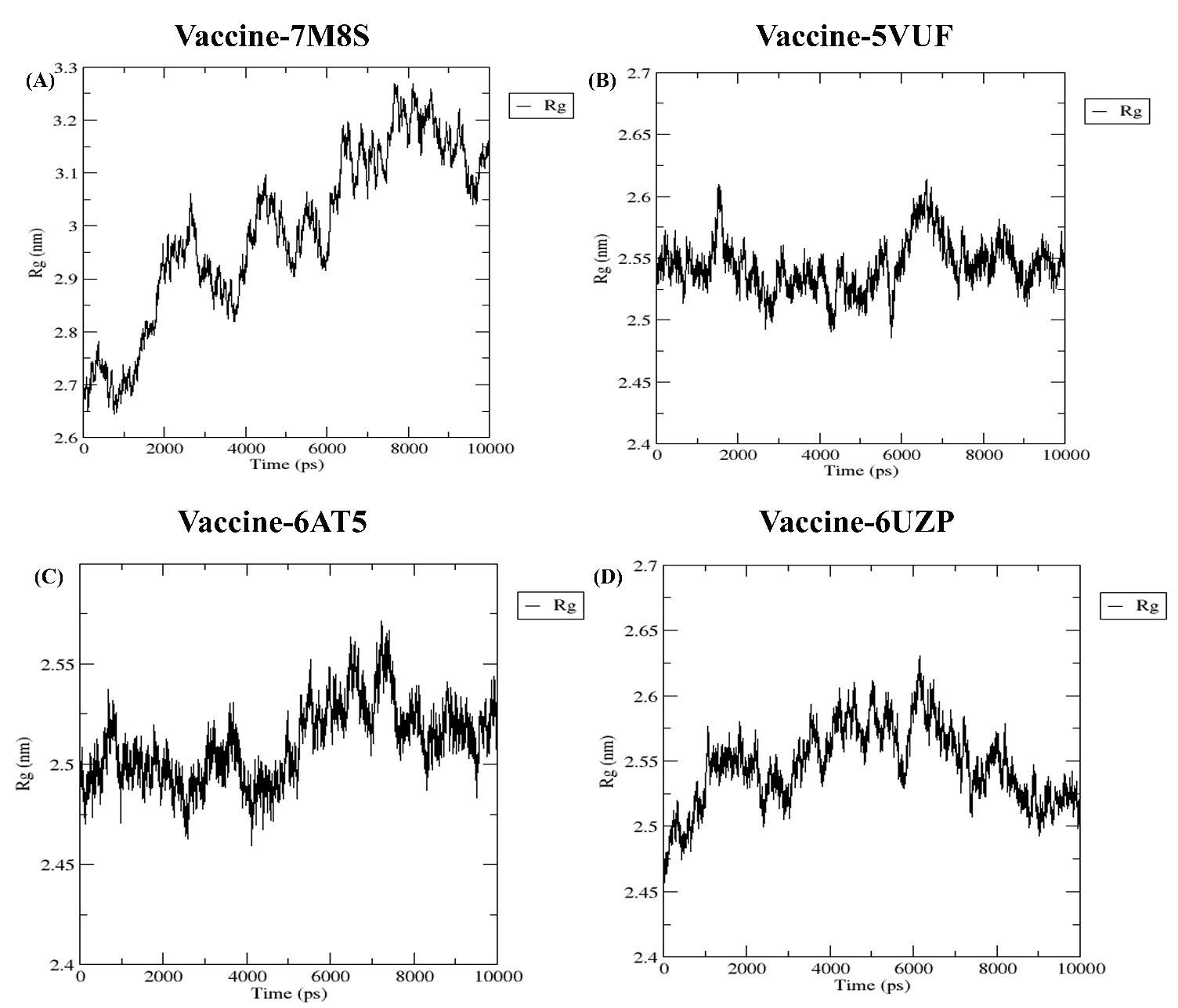
**

**Supplementary Figure S3: Radius of Gyration of the vaccine and the four HLA alleles docked through the HDock web server. A) VAccine-7M8S complex B) Vaccine-5VUF complex C) Vaccine-6AT5 D) Vaccine-6UZP complex**

**
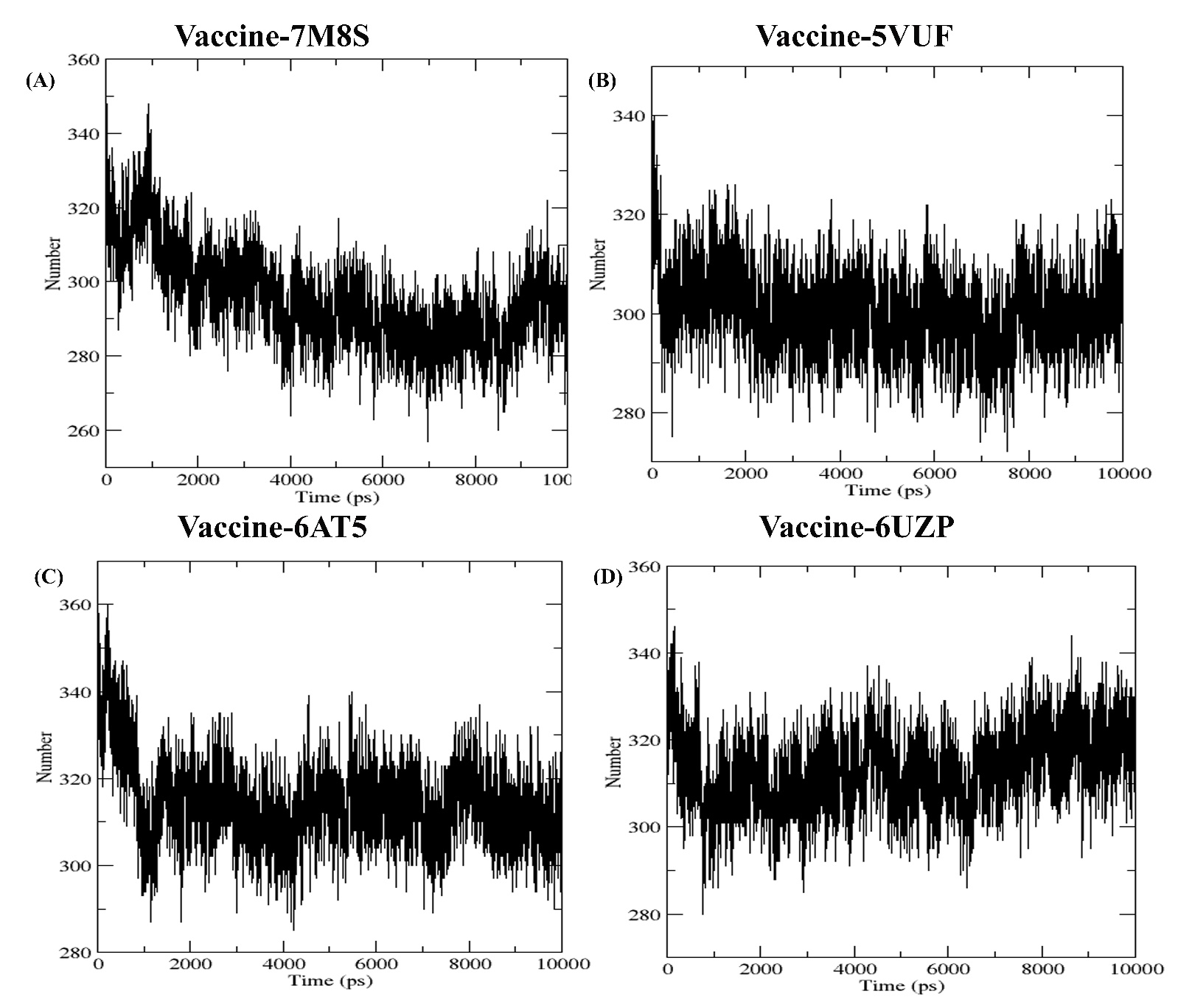
**

**Supplementary Figure S4: Hydrogen Bond of the vaccine and the four HLA alleles docked through the HDock web server. A) VAccine-7M8S complex B) Vaccine-5VUF complex C) Vaccine-6AT5 D) Vaccine-6UZP complex**

**
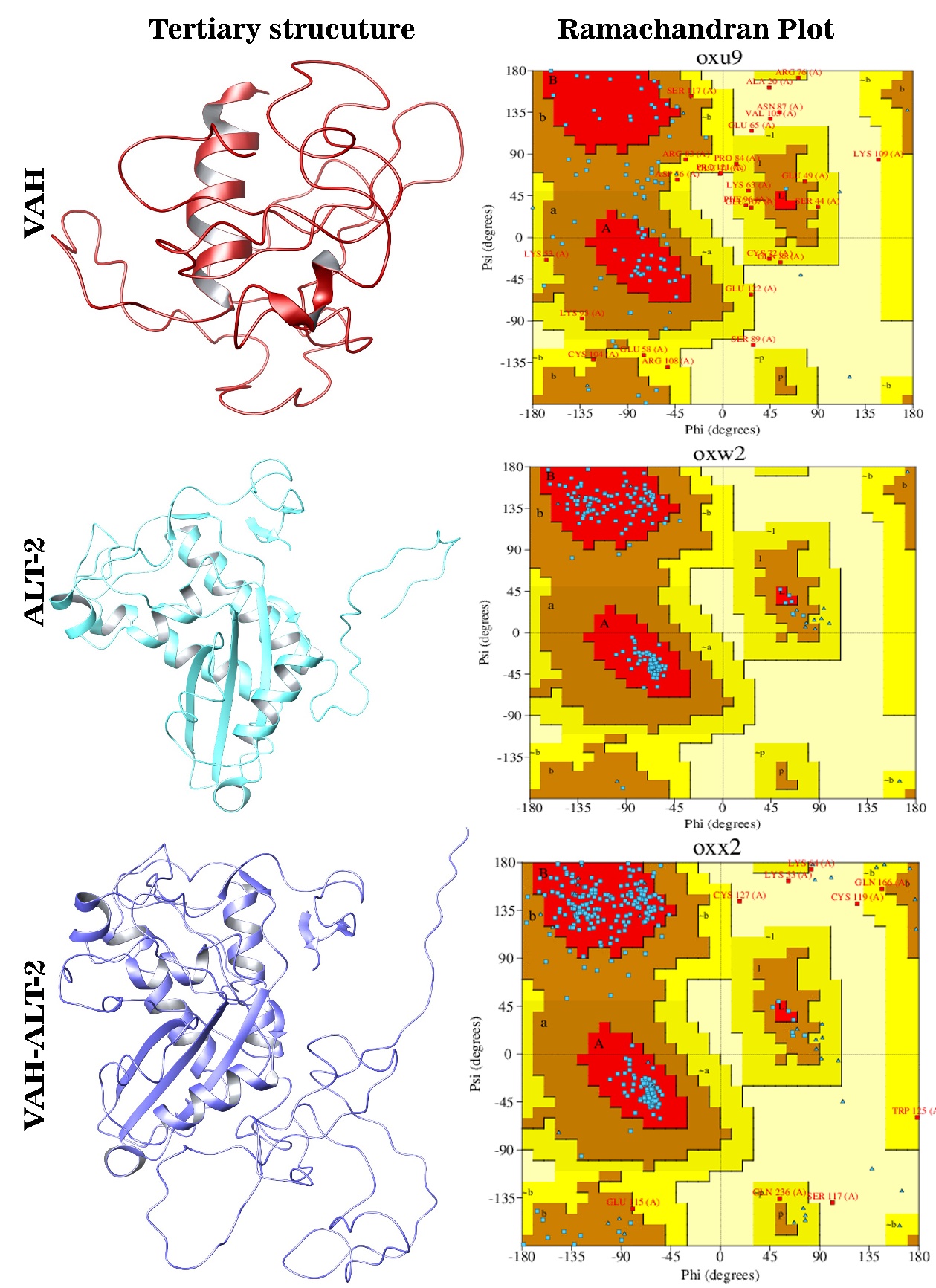
**

**Supplementary Figure S5:** Tertiary structure models of VAH, ALT-2 and fusion vaccine (VAH-ALT-2)
